# A Nearly Neutral Model of Molecular Signatures of Natural Selection after Change in Population Size

**DOI:** 10.1093/gbe/evac058

**Published:** 2022-04-27

**Authors:** Rebekka Müller, Ingemar Kaj, Carina F. Mugal

**Affiliations:** 1 Department of Mathematics, Uppsala University, 752 37 Uppsala, Sweden; 2 Department of Ecology and Genetics, Uppsala University, 752 36 Uppsala, Sweden

**Keywords:** nonequilibrium theory, nearly neutral theory, demographic nonequilibrium, theoretical population genetics, selection–drift balance

## Abstract

The nearly neutral theory is a common framework to describe natural selection at the molecular level. This theory emphasizes the importance of slightly deleterious mutations by recognizing their ability to segregate and eventually get fixed due to genetic drift in spite of the presence of purifying selection. As genetic drift is stronger in smaller than in larger populations, a correlation between population size and molecular measures of natural selection is expected within the nearly neutral theory. However, this hypothesis was originally formulated under equilibrium conditions. As most natural populations are not in equilibrium, testing the relationship empirically may lead to confounded outcomes. Demographic nonequilibria, for instance following a change in population size, are common scenarios that are expected to push the selection–drift relationship off equilibrium. By explicitly modeling the effects of a change in population size on allele frequency trajectories in the Poisson random field framework, we obtain analytical solutions of the nonstationary allele frequency spectrum. This enables us to derive exact results of measures of natural selection and effective population size in a demographic nonequilibrium. The study of their time-dependent relationship reveals a substantial deviation from the equilibrium selection–drift balance after a change in population size. Moreover, we show that the deviation is sensitive to the combination of different measures. These results therefore constitute relevant tools for empirical studies to choose suitable measures for investigating the selection–drift relationship in natural populations. Additionally, our new modeling approach extends existing population genetics theory and can serve as foundation for methodological developments.

**Significance**

A central question in evolutionary genetics concerns the relative contribution of natural selection versus chance to evolution, but theoretical predictions and empirical observations do not always provide a congruent picture about this question. Our hypothesis for this ambiguity is that theoretical predictions usually rely on equilibrium assumptions while most natural populations are not in equilibrium. To investigate this hypothesis, we formulate a mathematical framework for a demographic nonequilibrium scenario, which enables us to reconcile theory and data and also can serve as a practical guide on study design and interpretation of empirical observations.

## Introduction

Among the key driving factors of evolution are mutations, natural selection, and genetic drift. The analysis of the interplay between them provides valuable understanding on the genetic variation within and among populations and on their ability to evolve and adapt. Population genetics theory provides a mathematical approach to describe and analyse the interaction of the population-level processes. In particular, such theory predicts that the strength of genetic drift is weaker in larger populations than in smaller populations, due to the stochastic nature of reproduction (Wright 1931; Kimura 1964). This results in a positive correlation between the efficacy of selection and population size, the selection–drift balance. As a consequence, the rate of molecular evolution is influenced by the population size, in particular in the presence of weakly selected mutations (Kimura 1964; Ohta 1973, 1976).

The nearly neutral theory of molecular evolution emphasizes the importance of weakly selected mutations on a genome-wide scale (Ohta 1973, 1976, 1992). Within this framework, typically the distribution of fitness effects (DFE) of new mutations is weighted towards purifying selection: most mutations are deleterious, of which a nonnegligible amount is slightly deleterious, and only a small proportion of mutations is advantageous. The smaller the population size the more (deleterious) mutations fall into the weak selection regime, potentially contributing to segregating polymorphisms and fixation due to genetic drift. These molecular signatures make it possible to investigate the predictions of the nearly neutral theory in empirical studies with help of genomic data.

**Fig. 1.**
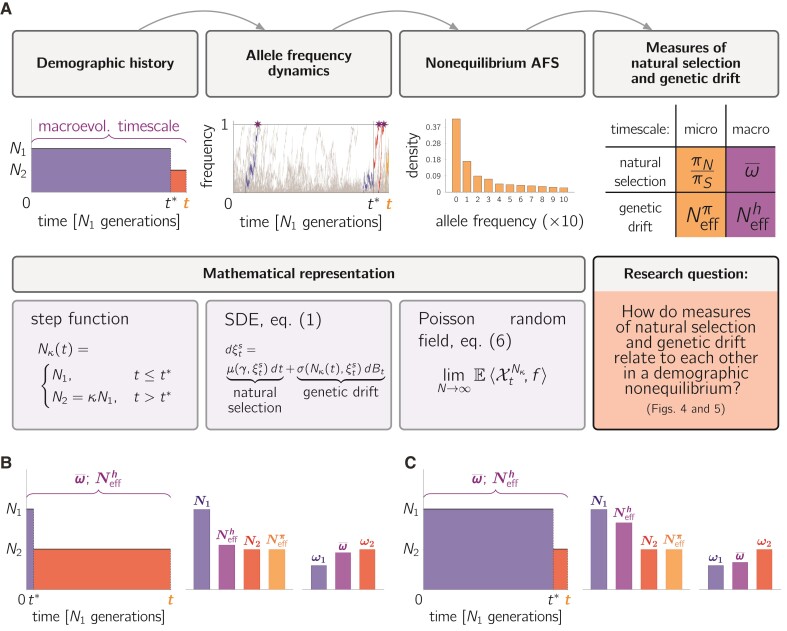
Study design and research question. Panel *A*: Illustration of the workflow. (i) A single change in population size at time $t^{*}$ is modeled by a step function. We visualize the macroevolutionary timescale that spans the time interval [0,t] (indicated in purple), and the microevolutionary timescale that provides a snapshot at time *t* (indicated in orange). (ii) We model the impact of demographic history on the allele frequency dynamics in [0,t] as the solution to the stochastic differential equation stated in equation (1). Different categories of allele frequency trajectories can be distinguished. Representative trajectories are highlighted in the respective color coding: mutations that segregate before $t^{*}$ (blue), mutations that arise before but continue to segregate after $t^{*}$ (blue-red), mutations that arise and segregate after $t^{*}$ but no longer at time *t* (red), mutations resulting in polymorphisms segregating at time *t* (orange). Representative trajectories that result in fixations and accumulate over [0,t] are highlighted by stars. (iii) A snapshot of the population dynamics at any point in time is described by the distribution of allele frequencies at that specific point in time and is summarized in the AFS. (iv) Based on the AFS we are able to derive different measures of natural selection and genetic drift at any point in time. From this the main question arises as how these measures relate to each other in a demographic nonequilibrium? Panels *B* and *C*: Examples of an ancient (*B*) and more recent (*C*) change in population size and their impact on measures of $N_{\mathrm{eff}}$ and the fixation rate ratio $\bar{\omega }$. (For the color representation of this figure the reader is referred to the online version of this paper.)

To detect evidence of selection in genome data, different approaches and methods have been developed (reviewed in Nielsen 2005; Vitti et al. 2013; Booker et al. 2017). A common feature of quantitative methods is to contrast neutral reference and test data, such as the contrast between synonymous and nonsynonymous mutations in protein-coding sequences. Here, we can distinguish between measures of natural selection at the micro- and macroevolutionary timescale (Vitti et al. 2013). Measures at the microevolutionary timescale, which are designed to identify selective events within a species, are typically based on segregating polymorphisms and give a *snapshot* of the current state. A popular representative is the ratio of nonsynonymous and synonymous diversity, $\pi_{N}/\pi_{S}$ (Nei and Li 1979). Macroevolutionary measures assess lineage-specific selection over larger evolutionary timescales in a phylogenetic setting. These measures are *accumulative* and typically based on interspecific differences that result from fixations in one lineage after divergence from a common ancestor. A measure that belongs to this group is the ratio of the nonsynonymous and synonymous sequence divergence, $d_{N}/d_{S}$ (Goldman and Yang 1994; Muse and Gaut 1994), which represents an estimate of the ratio of nonsynonymous and synonymous fixations in the time period after species divergence (Mugal et al. 2020). While the instantaneous fixation rate ratio is frequently denoted as ω, we introduce notation $\bar{\omega }$ for the ratio of nonsynonymous and synonymous fixations after species divergence in order to emphasize its accumulative character (fig. 1*A*).

The traditional approach to assessing genetic drift is to apply some version of an effective population size, $N_{\mathrm{eff}}$. Conceptually, $N_{\mathrm{eff}}$ relates a given (nonideal) population with a simpler idealized reference model, such as the ideal Wright–Fisher model, with respect to a particular property. This leads to different definitions of $N_{\mathrm{eff}}$, e.g. inbreeding and variance (Wright 1931; Crow and Kimura 1970) or eigenvalue effective size (Ewens 1979). In addition, also life-history traits are frequently used as proxies for $N_{\mathrm{eff}}$ (Nikolaev et al. 2007; Lee et al. 2011; Waples et al. 2013; Figuet et al. 2016; Bolívar et al. 2019). All approaches predict $N_{\mathrm{eff}}$ under different circumstances as for example certain spatial and temporal scales and demographic scenarios. Often it is not evident whether underlying assumptions of the various models are met in natural populations and how accurate the resulting estimates of $N_{\mathrm{eff}}$ are in case assumptions are violated. For this reason, the spatial and temporal scales of different estimates of $N_{\mathrm{eff}}$ have to be interpreted carefully to draw firm conclusions (Otto and Whitlock 1997; Wang et al. 2016; Nadachowska-Brzyska et al. 2021).

The nearly neutral theory predicts a negative correlation between $N_{\mathrm{eff}}$ and the measures $\pi_{N}/\pi_{S}$ and $\bar{\omega }$. However, this prediction is based on the equilibrium assumption, where the effect of genetic drift on segregating polymorphisms balances the efficacy of selection implying a constant evolutionary rate. Yet, changes in population size, amongst other factors, generally cause a nonequilibrium for a prevalent amount of time, which disturbs the selection–drift balance (Brandvain and Wright 2016). In a meta-analysis, Brandvain and Wright (2016) compare predictions of classical (equilibrium) theory with results from a large number of empirical studies. This analysis stresses the need of care for nonequilibrium conditions when evaluating differences in selection efficacy among species. To enable such care to be taken, simulation studies and mathematical models are critical tools to investigate the effects of demographic nonequilibria on different evolutionary processes. Simulation-based studies are able to generate observational insight of complex scenarios, such as fluctuating population sizes and the effect of linked selection in demographic nonequilibria (Rousselle et al. 2018; Torres et al. 2020). A strength of mathematical approaches is the ability to clearly decompose effects of nonequilibrium conditions on the processes driving evolution. This constitutes a valuable complement to simulation studies and in turn provides the possibility to develop refined methodology, compare e.g. Evans et al. (2007), Živković and Stephan (2011), Živković et al. (2015) and Kaj and Mugal (2016).

In this study, we investigate the effect of a single change in population size on micro- and macroevolutionary measures of selection in an otherwise ideal population (fig. 1). Concentrating on this isolated aspect enables us to derive exact analytical results that are straightforward to interpret. In a pioneering work, Eyre-Walker (2002) addressed the isolated scenario of a change in population size with help of the stationary Poisson random field framework. In this setting, the nonequilibrium is modeled only indirectly as the weighted sum of the ancestral and the new equilibrium value. This original model forms the basis for many methodological developments (Keightley and Eyre-Walker 2007; Charlesworth and Eyre-Walker 2008; Eyre-Walker and Keightley 2009; Schneider et al. 2011; Kousathanas and Keightley 2013), which have found wide application in evolutionary genetic studies. Nevertheless, as a consequence of the stationarity assumption, the effects of a demographic nonequilibrium on allele frequency trajectories are ignored (Williamson et al. 2005; Boyko et al. 2008).

Here, we explicitly model the impact of a change in population size on allele frequency trajectories. Specifically, we build on the Poisson random field framework approach as in Kaj and Mugal (2016) and derive the nonstationary allele frequency spectrum (AFS) after a change in population size. This enables us to obtain time-dependent formulations of the above addressed measures, $\left(\pi_{N}/\pi_{S})(t\right)$ and $\bar{\omega }(t)$. The study setup, connected to the mathematical framework, is illustrated in figure 1. The time-dependent formulations allow for the discussion of the following questions: First, how does a change in population size affect micro- and macroevolutionary measures of natural selection, $\left(\pi_{N}/\pi_{S})(t\right)$ and $\bar{\omega }(t)$? Second, how does a change in population size affect the relationship between measures of natural selection and genetic drift during the nonequilibrium period? To this end, we investigate different choices of $N_{\mathrm{eff}}$ as measures of genetic drift. Finally, we discuss the relevance of micro- and macroevolutionary measures for empirical studies of the selection–drift relationship and outline possible applications and extensions of the model.

## Results

### Basic Model

Our goal is to formulate a mathematical model that describes the allele frequency evolution in a population during a time interval in which the population experiences a change in population size. Within this framework, we shall then derive an analytical description of the nonequilibrium AFS, which will enable us to study the behavior of micro- and macroevolutionary measures of natural selection in a nonequilibrium population (fig. 1*A*). Specifically, we consider the allele frequency evolution in a population that undergoes an instantaneous change in population size at a single point in time $t^{*}$ from constant size *N* to constant size κN, where κ is a positive parameter. In other words, the population size over time is a step function $N_{\kappa }$ such that $N_{\kappa }(t)=N$, $t<t^{*}$, and $N_{\kappa }(t)=\kappa N$, $t\geq t^{*}$, compare figure 1 where $N_{1}\;\hat{=}\;N$ and $N_{2}\;\hat{=}\;\kappa N$.

Throughout this work, we use *N* as reference size and apply an evolutionary timescale where one unit of time corresponds to [N] generations. We will consider a time interval [0,t], with *t* corresponding to the present time, and 0 corresponding to a point in time [Nt] generations in the past. We then examine a population that undergoes an ancient change in population size at $t^{*}$ close to 0 (fig. 1*B*), and a population that undergoes a more recent change at $t^{*}$ close to *t* (fig. 1*C*). For generality, we let *N* represent the population size of a haploid population. Under the assumption of additive fitness effects in a diploid organism, an assumption common to many methodological developments in the field (Keightley and Eyre-Walker 2007; Charlesworth and Eyre-Walker 2008; Eyre-Walker and Keightley 2009; Schneider et al. 2011; Kousathanas and Keightley 2013; Johri et al. 2020), this is equivalent to a diploid population of size N/2. Each haploid individual is characterized by a genome sequence of *L* independent sites, which corresponds to the assumption of free recombination across sites. Random mutations arrive independently and uniformly over individuals on monomorphic sites with population mutation intensity θ per generation in the reference population. Hence, as long as $t<t^{*}$, the mutation intensity per time unit is θN. Consequently, for $t\geq t^{*}$, the mutation intensity is κθ per generation and κθN per time unit. Since a mutation arises in a single individual, its initial frequency is $1/N_{\kappa }$, i.e. 1/N or 1/(κN) dependent on if it arises before or after the change in population size. Each mutation is assigned a population selection intensity γ.

We use the Wright–Fisher model with selection (Fisher 1930; Wright 1931) for two alleles segregating at one site to model reproduction and then study the population dynamics of the collection of all *L* independent sites. In the limit as *L* tends to infinity and *N* is large but fixed, the number of new mutations over all mono-allelic sites is approximately Poisson distributed with mean $\theta N_{\kappa }$ per time unit (Kaj and Mugal 2016). When taking N→∞ the initial frequency of new mutations, $1/N_{\kappa }$, balances the mutational input and ensures that it does not become infinite. Under these limits, the Poisson random field approximation applies (Sawyer and Hartl 1992; Kaj and Mugal 2016). The derived allele frequencies are independent over polymorphic sites. The allele frequency in a single site starting at time *s* evolves as a Wright–Fisher diffusion process with selection, that is, a solution of the stochastic differential equation

(1) $$d\xi_{t}^{s}=\;\gamma \xi_{t}^{s}(1-\xi_{t}^{s})\;dt+\sqrt{\frac{1}{N_{\kappa }(t)/N}\xi_{t}^{s}(1-\xi_{t}^{s})}\;dB_{t},\;t\geq s,$$

with initial value $\xi_{s}^{s}=y\in (0,1)$—typically $1/N_{\kappa }(s)$. Here, $B_{t}$ is a standard Brownian motion and $N_{\kappa }(t)/N=1$ whenever $t<t^{*}$ and $N_{\kappa }(t)/N=\kappa$ for $t\geq t^{*}$. The Brownian motion part of the equation encodes genetic drift that varies depending on the population size. Basically, equation (1) describes that the frequency of a mutant allele changes randomly but is pushed towards 1 (fixation) or 0 (extinction) depending on the selection coefficient. We denote such a Markov process by $(\xi_{t}^{s}{)}_{t\geq s}$ or simply $(\xi_{t}{)}_{t\geq 0}$ when the initial time is s=0. Furthermore, let $P_{y}^{\gamma ,\kappa }$ and $E_{y}^{\gamma ,\kappa }$ be the law and expectation of processes $(\xi_{t}{)}_{t}$ that start in *y*, have selective pressure γ, and evolve in a population of size κN. Let $\tau_{1}$ be the time to fixation of the derived allele. Hence, the fixation probability for a derived allele with frequency *y* and selective pressure γ is given by (Kimura 1962)

(2) $$\begin{array}{rl} & q_{\gamma ,\kappa }(y)=P_{y}^{\gamma ,\kappa }(\tau_{1}<\infty )=\frac{1-e^{-2\gamma \kappa y}}{1-e^{-2\gamma \kappa }},\;\gamma \neq 0,\\ & q_{0,\kappa }(y)=y \end{array}$$

for a fixed κ. As N→∞, the scaled fixation rate emerges as

(3) $$\begin{array}{rl} & Nq_{\gamma ,\kappa }(1/N)\to \omega_{\gamma ,\kappa }=\frac{2\gamma \kappa }{1-e^{-2\gamma \kappa }},\;\gamma \neq 0,\\ & \omega_{0,\kappa }=1. \end{array}$$

This means, in an equilibrium population of size κN, the instantaneous fixation rate ratio of a class of selected (with selective pressure γ) and neutral mutations in the limit equals $\omega_{\gamma ,\kappa }$.

Returning to the Poisson random field setting, the allele frequencies are represented by Poisson points $\left(s,\xi^{s}\right)$ on the collection of sites according to the Poisson distribution with intensity $\theta N_{\kappa }$: once such a mutation event takes place at a certain time *s*, a path $(\xi_{t}^{s}{)}_{t\geq s}$ is initialized at frequency $1/N_{\kappa }(s)$. We fix $t^{*}>0$ and represent the state of the Poisson random field, i.e. the collection of allele frequencies, at time *t* as a random measure $X_{t}^{N_{\kappa }}(dy)$ on (0,1]. We further focus on the allele frequency evolution for t>0, i.e. we will ignore fixations for t≤0 but start from polymorphic frequencies on (0,1) at t=0. A visualization of the setup is presented in figure 1*A* (ii). It is known that the aggregate of all mutations from the infinite past in the ancestral population builds up a Poisson measure in steady state (Kaj and Mugal 2016). More precisely, the relevant initial distribution of allele frequencies at t=0 for our model, that is $X_{0}^{N_{\kappa }}(dy)$, is a Poisson measure with intensity measure $\omega_{\gamma ,1}\;\psi_{\gamma ,1}(y)\;dy$ on (0,1), where

(4) $$\psi_{\gamma ,\kappa }(y)=\frac{1-e^{-2\gamma \kappa (1-y)}}{\gamma y(1-y)},\;\gamma \neq 0,\;\psi_{0,\kappa }(y)=\frac{2\kappa }{y}.$$

The initial distribution of trajectories at t=0 plus the arrival of new mutations during (0,t] together preserve the Poisson distribution which is invariant as long as the population size does not change, i.e. for $0<t\leq t^{*}$. To account for fixations during [0,t] we also include the singular contribution at y=1, $X_{t}^{N_{\kappa }}(\{1\})$, t≥0, which is a Poisson counting process with time-inhomogeneous intensity. Using suitable functions *f*, the evaluation $\left\langle X_{t}^{N_{\kappa }},f\rangle =\sum_{i}f(y_{i}\right)$ is the sum over the random number of segregating sites present in the population at time *t* and keeps track of the corresponding allele frequencies $y_{i}$. The expected value $EX_{t}^{N_{\kappa }}$ is a deterministic measure on the frequency interval [0,1], which in the limit N→∞ of $E\langle X_{t}^{N_{\kappa }},f\rangle$ allows for the interpretation of allele frequency spectrum.

We discuss the formal construction of the random measure model $X_{t}^{N_{\kappa }}$ in “Materials and Methods”. Details of the presentation and most of the technical aspects are deferred to the Supplementary Sections 1.1 and 1.2, Supplementary Material online.

### Nonequilibrium Allele Frequency Spectrum

The AFS accounts for the collection of all derived allele frequencies across sites at a fixed point in time. More formally, the spectrum of allele frequencies *y*, 0<y<1, represents the average intensity of attained frequency values at *t*, $\xi_{t}^{s}=y$ for some s≤t, compare figure 1*A*. In our approach, we also include alleles which have reached fixation during [0,t]. As a reference case we begin with the equilibrium AFS, which arises as the scaled limit of expected values for the case of a fixed size population, say κN,

(5) $$\lim_{N\to \infty }E\langle X_{t}^{\kappa N},f\rangle =\theta \omega_{\gamma ,\kappa }f(1)t+\theta \int_{0}^{1}f(y)\omega_{\gamma ,\kappa }\psi_{\gamma ,\kappa }(y)\;dy,$$

for suitable functions *f* satisfying sufficient conditions for these integrals to be well defined. The linear term in *t* represents the effect of constant rate fixations and the integral term independent of time represents the steady-state spectrum of polymorphic frequencies.

Now, considering a population undergoing a change in size at time $t^{*}$, equation (5) applies with κ=1 as long as $0<t\leq t^{*}$. It is only when we attempt to extend relation (5) beyond $t^{*}$ that the change in population size begins to alter the composition of weights of allele frequencies. The collection of paths at a time point $t>t^{*}$ contains both ancestral trajectories of alleles which were present already at $t^{*}$ and new paths emerging from mutations taking place subsequent to the change in population size. The additional contributing terms together with those in relation (5) yield

(6) $$\begin{array}{rll} \lim_{N\to \infty }E\langle X_{t}^{N_{\kappa }},f\rangle & = & \theta f(1)[\omega_{\gamma ,1}t^{*}+\omega_{\gamma ,\kappa }(t-t^{*})]\\ & & +\theta \int_{0}^{1}f(y)\omega_{\gamma ,\kappa }\psi_{\gamma ,\kappa }(y)\;dy\\ & & +\theta \int_{0}^{1}E_{y}^{\gamma ,\kappa }[f(\xi_{t}^{t^{*}})][\omega_{\gamma ,1}\psi_{\gamma ,1}(y)\\ & & -\omega_{\gamma ,\kappa }\psi_{\gamma ,\kappa }(y)]\;dy, \end{array}$$

in detail derived in the Supplementary Section 1, Lemma 3(i) and Theorem 1, Supplementary Material online. While in this representation we do not see directly a spectrum of frequencies *y* with explicit weights affecting f(y), we do see indirectly the time-dependence effect due to the nonequilibrium framework.

The mathematical framework presented in this work permits retrieving time-dependent expressions for relevant summary statistics by application of selected functions *f* to the nonequilibrium AFS in equation (6). In this sense we consider nucleotide diversity associated with the function $f_{\mathrm{pw}}(y)=2y(1-y)$ and fixation rate associated with $f_{\mathrm{fix}}=1_{\left\{1\right\}}(y)$, as well as their respective ratios for nonsynonymous and synonymous mutations (see “Materials and Methods” for details).

### Measures of Natural Selection in a Nonequilibrium Population

**Fig. 2.**
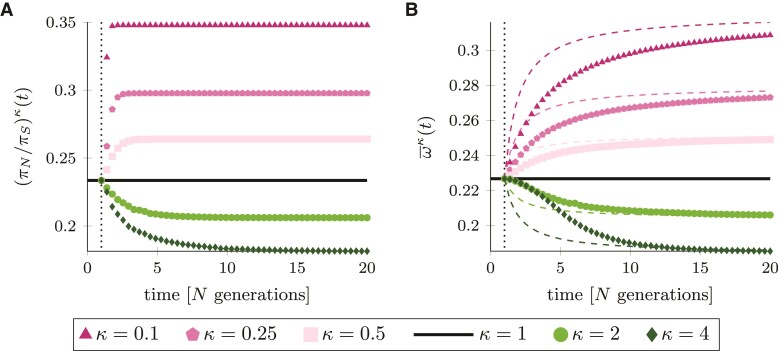
Measures of selection for different values of κ as functions of time. Panel *A*: the ratio of nonsynonymous and synonymous diversity $\left(\pi_{N}/\pi_{S}{)}^{\kappa }(t\right)$. Panel *B*: the fixation rate ratio ${\bar{\omega }}^{\kappa }(t)$. For comparison, colored, dashed curves represent the weighted fixation rate ratio $\omega_{w}^{\kappa }(t)$. Vertical, dotted lines indicate time $t^{*}=1$. Parameters: θ=1, and a=0.15 and ab=2500 for the DFE.

We study the behavior of two molecular measures of natural selection as functions of time after a change in population size, that is the ratio of nonsynonymous and synonymous genetic diversity, $\left(\pi_{N}/\pi_{S})(t\right)$, and the ratio of nonsynonymous and synonymous fixations, $\bar{\omega }(t)$, over a time interval [0,20]. In this setting, t=20 corresponds to the present time and t=0 to a time 20N generations in the past. A change in population size occurs at $t^{*}=1$, i.e. 19N generations in the past, which we refer to as ancient change (fig. 1*B*). Since we are particularly interested in the prediction of the nearly neutral theory in nonequilibrium, we consider a DFE restricted to deleterious mutations ranging from strongly to slightly deleterious fitness effects approximated by a Γ-distribution. Figure 2*A* shows the behavior of $\left(\pi_{N}/\pi_{S}{)}^{\kappa }(t\right)$ for different extents and directions of change in population size, κ∈{0.1,0.25,0.5,1,2,4}. The time it takes to reach the new equilibrium depends on both, the direction and extent of change in population size: the new equilibrium is reached more quickly in case of a population decline (κ<1, the larger the reduction the faster). For an increase in population size (κ>1), it takes longer to attain the new equilibrium. Also, given a DFE restricted to deleterious mutations, $\left(\pi_{N}/\pi_{S}{)}^{\kappa }(t\right)$ is negatively correlated with population size as predicted by the nearly neutral theory of molecular evolution. The behavior of ${\bar{\omega }}^{\kappa }(t)$ after a change in population size is depicted in figure 2*B* (and fig. 1*B*) and resembles the behavior of $\left(\pi_{N}/\pi_{S}{)}^{\kappa }(t\right)$. The ratio decreases for κ>1, which means that fewer deleterious nonsynonymous mutations reach fixation—in accordance with observations about selection acting more efficiently in larger populations. However, ${\bar{\omega }}^{\kappa }(t)$ is an accumulative measure over the time interval [0,t], while $\left(\pi_{N}/\pi_{S}{)}^{\kappa }(t\right)$ reflects a snapshot of the strength of selection at time *t*. As a consequence, it takes longer for ${\bar{\omega }}^{\kappa }(t)$ to reach its new equilibrium than it does for $\left(\pi_{N}/\pi_{S}{)}^{\kappa }(t\right)$.

Another means to capture the impact of a change in population size on ${\bar{\omega }}^{\kappa }(t)$ is to consider the weighted sum of the ancestral and the new equilibrium value (Eyre-Walker 2002). In our notation, this reads $\omega_{w}^{\kappa }(t)\;:=[t^{*}\omega^{1}+(t-t^{*})\omega^{\kappa }]/t$ for equilibrium instantaneous fixation rates $\omega^{1}$ and $\omega^{\kappa }$, respectively. To visualize the difference between ${\bar{\omega }}^{\kappa }(t)$ and $\omega_{w}^{\kappa }(t)$, the weighted fixation rate ratios are included as dashed lines in figure 2*B*. The nonequilibrium model derived in this study shows that the function ${\bar{\omega }}^{\kappa }(t)$ reacts more slowly to the change in population size and takes longer to reach the new equilibrium value in comparison to the approach of weighting the equilibrium values. This illustrates that ignoring the period where allele frequencies are in nonequilibrium, as for example implemented in methods to estimate the DFE (Keightley and Eyre-Walker 2007; Eyre-Walker and Keightley 2009; Schneider et al. 2011; Kousathanas and Keightley 2013), leads to an underestimation of the time until ${\bar{\omega }}^{\kappa }(t)$ reaches its equilibrium.

Note that figure 2*B* shows the fixation rate ratio for a change in population size at time $t^{*}=1$. Changes at other time points can lead to severely different behaviors. A change at $t^{*}\leq 0$, for example, would lead to $\omega_{w}^{\kappa }(t)=\omega^{\kappa }$ without reflecting any influence of the ancestral population. On the other hand, if the change in population size happens more recently in time (fig. 1*C*), the contribution of the ancient population size becomes more pronounced (see Supplementary fig. S1, Supplementary Material online). In addition, we note that $\bar{\omega }(t)$ represents a population functional, that assesses fixations in the whole population or lineage. The common estimate of $\bar{\omega }(t)$ is $d_{N}/d_{S}$, which represents a sample functional and introduces further bias for small *t*, but converges for t→∞ (Mugal et al. 2014, 2020).

### Proxies of Effective Population Size as Measures of Genetic Drift

In order to evaluate the prediction of the nearly neutral theory in nonequilibrium we need to relate the above-introduced measures of selection to estimates of the effective population size. Since there are various ways to define $N_{\mathrm{eff}}$, it is fundamental to first discuss the differences and to assess which of the definitions are relevant to relate to $\left(\pi_{N}/\pi_{S}{)}^{\kappa }(t\right)$ and ${\bar{\omega }}^{\kappa }(t)$ in our modeling approach. The most commonly considered concepts of $N_{\mathrm{eff}}$ among others are variance and inbreeding (Wright 1931, 1940; Crow 1954; Crow and Kimura 1970), coalescent (Lynch and Conery 2003), and eigenvalue effective population size (Ewens 1969, 1979, 1982). The properties, that these concepts aim to model, are the variance in allele frequencies over time due to random genetic drift, the average inbreeding coefficient, the rate of coalescence of neutral alleles, and the leading nonunit eigenvalue of the allele frequency transition matrix.

We here focus on the pairwise synonymous nucleotide diversity (Lynch and Conery 2003; Wakeley and Sargsyan 2008; Ellegren and Galtier 2016) and the harmonic mean effective population size over [0,t] (Wright 1940; Karlin 1968; Nei and Tajima 1981). The scaled pairwise synonymous diversity, $N_{\mathrm{eff}}^{\pi }(t)\;:=\pi_{S}^{\kappa }(t)/(2L\mu )$, where μ:=θ/(LN) is the mutation rate per generation and individual, is an estimate of effective population size based on genetic variation and accordingly represents a microevolutionary measure of effective population size. We note that scaled pairwise synonymous diversity is often also perceived as coalescent effective population size (Lynch and Conery 2003; Wakeley and Sargsyan 2008).

The harmonic mean effective size over [0,t] is a representative of variance effective population size and defined as the average of genetic drift over the time interval [0,t] with $t^{*}\in [0,t]$,

$$N_{\mathrm{eff}}^{h}(t)\;:={\left(\frac{1}{t}\int_{0}^{t}\frac{1}{N_{\kappa }(s)}\;ds\right)}^{-1}=\frac{\kappa Nt}{t^{*}(\kappa -1)+t},$$

with $N_{\mathrm{eff}}^{h}(t^{*})=N$, and $N_{\mathrm{eff}}^{h}(t)\to \kappa N$ for *t* large. This means the ancestral population size *N* loses its influence on $N_{\mathrm{eff}}^{h}(t)$ the further in the past the change took place. If κ is constant over [0,t], then $N_{\mathrm{eff}}^{h}(t)=\kappa N$. Also, in view of the genetic drift term in equation (1)—the variance term of the SDE—the parameter κ at time *t* multiplied by *N* can be interpreted as a snapshot of the variance effective population size at time *t*. The harmonic mean effective population size over the time interval [0,t] for *t* large, on the other hand, can be considered a representative of long-term effective population size.

**Fig. 3.**
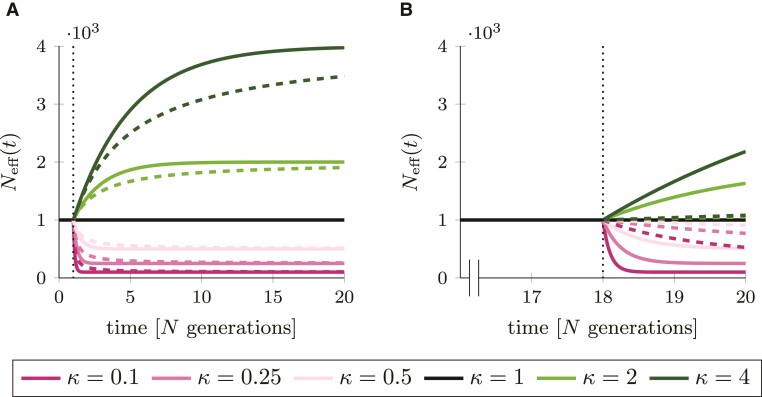
The effective population size based on nucleotide variation, $N_{\mathrm{eff}}^{\pi }(t)$ (solid lines), and the harmonic mean effective population size, $N_{\mathrm{eff}}^{h}(t)$ (dashed lines), for different values of κ. Black, dotted lines mark the time of change in population size, $t^{*}$. Panel *A* shows an *ancient* change in population size at $t^{*}=1$, panel *B* a *more recent* change at $t^{*}=18$. Parameters θ=1 and N=1000. (For the color representation of this figure the reader is referred to the online version of this paper.)

With the two measures of effective population size at hand, the microevolutionary measure $N_{\mathrm{eff}}^{\pi }(t)$ and the macroevolutionary measure $N_{\mathrm{eff}}^{h}(t)$, we investigate and compare how a change in population size is reflected in each of them. For this purpose, we consider two scenarios of change in population size: an ancient change at $t^{*}=1$, i.e. 19N generations in the past from present time t=20, (fig. 3*A*) and a more recent change at $t^{*}=18$, i.e. 2N generations in the past, (fig. 3*B*). For each scenario $N_{\mathrm{eff}}^{\pi }(t)$ (solid lines) and $N_{\mathrm{eff}}^{h}(t)$ (dashed lines) are plotted for different values of κ as functions of time.

For an ancient change, it seems that both proxies mirror the change in size to a large degree as they are close to the new equilibrium value. However, $N_{\mathrm{eff}}^{h}(t)$ reaches the new equilibrium value more slowly compared to $N_{\mathrm{eff}}^{\pi }(t)$. This holds in particular for κ>1, leading to the presumption that the more a population increases, the slower the new equilibrium is reached and vice versa. For a more recent change in population size, the difference between the two estimates is much more evident (fig. 3*B*). The proxy $N_{\mathrm{eff}}^{\pi }(t)$ responds quickly to a change in population size, as expected for a measure relevant at the microevolutionary timescale, while $N_{\mathrm{eff}}^{h}(t)$ is rather unaffected.

### The Selection–Drift Relationship After a Change in Population Size

**Fig. 4.**
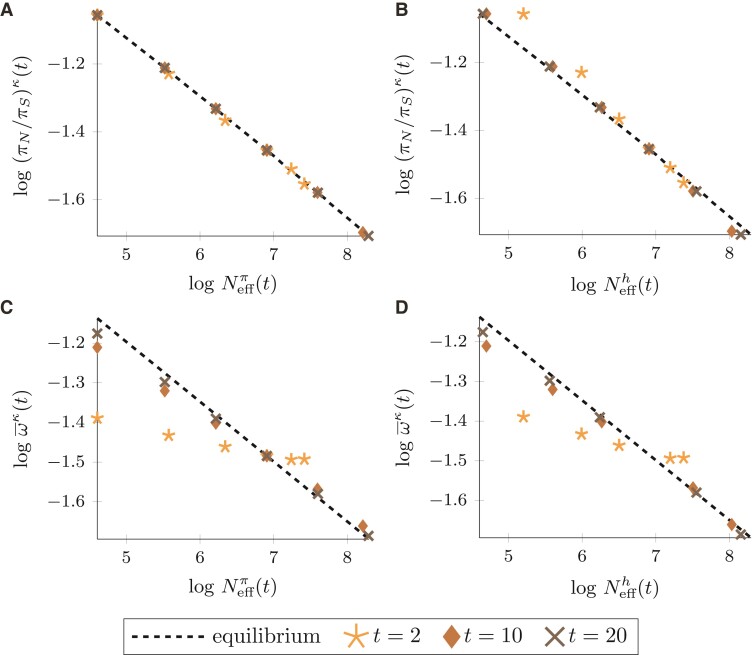
Proxies of $N_{\mathrm{eff}}$ versus measures of selection at time t∈{2,10,20} after an *ancient* change in population size at $t^{*}=1$ for κ∈{0.1,0.25,0.5,1,2,4}. Black, dashed lines show the expected relation in equilibrium populations. Panels *A* and *C*: genetic drift estimated by effective population size based on nucleotide variation, $N_{\mathrm{eff}}^{\pi }(t)$. Panels *B* and *D*: genetic drift estimated by the harmonic mean effective population size, $N_{\mathrm{eff}}^{h}(t)$. Parameters a=0.15 and ab=2500 in the DFE, N=1000, and θ=1.

**Fig. 5.**
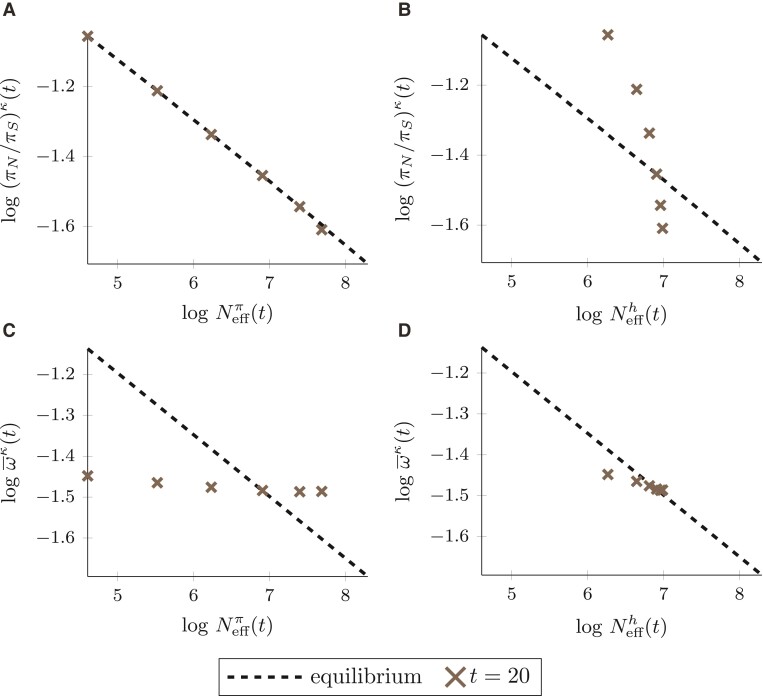
Proxies of $N_{\mathrm{eff}}$ versus measures of selection at time t=20 after a *more recent* change in population size at $t^{*}=18$ for κ∈{0.1,0.25,0.5,1,2,4}. Black, dashed lines show the expected relation in equilibrium populations. Panels *A* and *C*: genetic drift estimated by effective population size based on nucleotide variation, $N_{\mathrm{eff}}^{\pi }(t)$. Panels *B* and *D*: genetic drift estimated by the harmonic mean effective population size, $N_{\mathrm{eff}}^{h}(t)$. Parameters a=0.15 and ab=2500 in the DFE, N=1000, and θ=1.

We investigate the selection–drift relationship after a change in population size and compare it to the equilibrium behavior. For this purpose, we relate the ratio of nucleotide diversity, $\left(\pi_{N}/\pi_{S}{)}^{\kappa }(t\right)$, and the fixation rate ratio, ${\bar{\omega }}^{\kappa }(t)$, to the two measures $N_{\mathrm{eff}}^{\pi }(t)$ and $N_{\mathrm{eff}}^{h}(t)$, after an ancient ($t^{*}=1$, fig. 4) and a more recent ($t^{*}=18$, fig. 5) change in population size. To evaluate the nonequilibrium behavior, we indicate the expected relation of genetic drift and natural selection in equilibrium populations. For a fixed DFE following a Γ-distribution, the log–log relationship of the measures of selection at hand and proxies of $N_{\mathrm{eff}}$ is approximately linear at equilibrium (Kimura 1979; Welch et al. 2008),

$$\begin{array}{rl} \log\;(\pi_{N}/\pi_{S}) & ∼-a\log\;(N_{\mathrm{eff}})+C_{1},\\ \log\;(\bar{\omega }) & ∼-a\log\;(N_{\mathrm{eff}})+C_{2}, \end{array}$$

where the slope *a* is given by the shape parameter of the Γ-distribution and the intercept by some constants $C_{1}$ and $C_{2}$, respectively.

Figure 4 visualizes the selection–drift relationship at t=20 for an ancient change in population size at $t^{*}=1$, i.e. 19N generations in the past. In addition, the selection–drift relationship for t=2 and t=10 is shown to depict how the selection–drift relationship changes over time. When using $\left(\pi_{N}/\pi_{S}{)}^{\kappa }(t\right)$ as measure of natural selection (fig. 4*A* and *B*) a slight discrepancy between the prediction of the nearly neutral theory in equilibrium and the nonequilibrium behavior exists shortly after the change in population size, i.e. at t=2, but is not very pronounced. As the change in population size becomes more ancient, i.e. for t=10 and t=20, the discrepancy vanishes, regardless of the choice of measure for $N_{\mathrm{eff}}$. Using ${\bar{\omega }}^{\kappa }(t)$ as measure of natural selection (fig. 4*C* and *D*) also leads to a difference between equilibrium and nonequilibrium relationship for t=2. For an increase in population size, ${\bar{\omega }}^{\kappa }(t)$ is larger than expected in equilibrium, while the reverse is true for a population decline. This results in a flatter slope of the selection–drift relationship shortly after the change in population size. As time passes, i.e. for t=10 and t=20, the slope again approaches the equilibrium slope. Even though the linear approximation is less good during nonequilibrium, the deviations from linearity appear modest. The main difference between equilibrium and nonequilibrium is the slope of the selection–drift relationship, which in nonequilibrium, i.e. shortly after the change in population size, no longer is representative of the shape parameter of the DFE.

Since we see a clear deviation from the equilibrium prediction of the nearly neutral theory shortly after a change in population size, we also consider a more recent change at $t^{*}=18$ in figure 5, i.e. 2N generations in the past. Figure 5*A* shows that $\left(\pi_{N}/\pi_{S}{)}^{\kappa }(t\right)$ and $N_{\mathrm{eff}}^{\pi }(t)$ react quickly to the change in population size and their relationship closely follows the equilibrium behavior as both measures are affected similarly by the nonequilibrium. In contrast, a strong deviation from the equilibrium relationship is observed when relating $\left(\pi_{N}/\pi_{S}{)}^{\kappa }(t\right)$ to $N_{\mathrm{eff}}^{h}(t)$ as measure of genetic drift in figure 5*B*. A similarly strong deviation from the equilibrium relation but notably in the opposite direction is obtained when using the fixation rate ratio ${\bar{\omega }}^{\kappa }(t)$ as measure of selection and correlating it to $N_{\mathrm{eff}}^{\pi }(t)$ (fig. 5*C*). The deviations from the equilibrium selection–drift relationship in figure 5*B* and *C* clearly illustrate that the combination of microevolutionary and macroevolutionary measures is problematic, since microevolutionary measures react faster to a change in population size than macroevolutionary measures. If two macroevolutionary measures are related to each other, $N_{\mathrm{eff}}^{h}(t)$ and ${\bar{\omega }}^{\kappa }(t)$, the deviation from the equilibrium selection–drift relationship is less apparent, with both measures rather insensitive to more recent changes in population size (fig. 5*D*).

Overall, our analytical results clearly demonstrate that microevolutionary and macroevolutionary measures show different sensitivity to demographic events. As a consequence, the comparison of micro- and macroevolutionary measures of natural selection and genetic drift under ongoing demographic nonequilibria can essentially lead to a biased picture of the selection–drift relationship (fig. 5*B* and *C*). Also, depending on whether $\left(\pi_{N}/\pi_{S}{)}^{\kappa }(t\right)$ or ${\bar{\omega }}^{\kappa }(t)$ is considered, there is not only a difference in the degree of deviation, the slope of the log–log relationship changes into different directions. When comparing $\left(\pi_{N}/\pi_{S}{)}^{\kappa }(t\right)$ to a macroevolutionary measure of $N_{\mathrm{eff}}$ the slope is larger than in equilibrium (fig. 5*B*), while in case of ${\bar{\omega }}^{\kappa }(t)$ the slope is smaller than in equilibrium irrespective of what measure of $N_{\mathrm{eff}}$ is chosen (fig. 5*C* and *D*).

## Discussion

The key question of this study is how a change in population size affects the selection–drift balance. Our analytical results illustrate that in the absence of advantageous mutations the negative correlation between molecular measures of selection and genetic drift holds even during nonequilibrium periods. However, the strength of the relationship is clearly influenced during nonequilibrium periods and dependent on what measures of selection and $N_{\mathrm{eff}}$ are chosen for comparison. As a consequence, the slope of the log–log selection–drift relationship is no longer given by the shape parameter of the DFE.

### Implications for Empirical Evolutionary Genetics Studies

Our mathematical framework provides a guide to investigate the selection–drift relationship in a demographic nonequilibrium. Figures 4 and 5 suggest that it seems advisable to correlate microevolutionary measures of $N_{\mathrm{eff}}$ with microevolutionary measures of selection and macroevolutionary measures of $N_{\mathrm{eff}}$ with macroevolutionary measures of selection. These combinations will ensure that the influence of nonequilibrium periods is of similar extent on both, measures of selection and $N_{\mathrm{eff}}$, such that the slope of the log–log selection–drift relationship approximately reflects the shape parameter of the underlying DFE. Alternatively, our mathematical framework could also form the basis for methodological developments that directly account for the demographic nonequilibrium and thereby enable the combination of micro- and macroevolutionary measures. In addition, we can conclude that the observed selection–drift relationship based on common measures of selection and $N_{\mathrm{eff}}$ is in particular sensitive to the choice of measures for a more recent but not so much for an ancient change in population size, since for an ancient change both micro- and macroevolutionary measures have had sufficient time to equilibrate (fig. 4).

In empirical studies, the harmonic mean effective population size, $N_{\mathrm{eff}}^{h}$, is rather rarely used as proxy of long-term $N_{\mathrm{eff}}$. Instead life-history traits, such as body mass, propagule size, or longevity, find wide application for investigating the selection–drift relationship (Nikolaev et al. 2007; Popadin et al. 2007; Lartillot and Poujol 2010; Nabholz et al. 2013; Romiguier et al. 2014; Chen et al. 2017; Bolívar et al. 2019; Kutschera et al. 2020), since they are accessible for a wide range of species. The observed relationship between life-history traits and macroevolutionary measures of selection is frequently in line with the nearly neutral prediction of a negative correlation between measures of selection and $N_{\mathrm{eff}}$ (Nikolaev et al. 2007; Popadin et al. 2007; Lartillot and Poujol 2010; Nabholz et al. 2013; Romiguier et al. 2014; Bolívar et al. 2019). Also studies that correlate life-history traits with microevolutionary measures of selection obtain results consistent with this prediction (Brandvain et al. 2013; Slotte et al. 2013; Burgarella et al. 2015; Chen et al. 2017; Kutschera et al. 2020), in particular for the case where population size has been relatively stable over time. However, evaluation of the slope is complicated by the abstract nature of $N_{\mathrm{eff}}$. Moreover, as predicted by our study, if a population has undergone a more recent change in size, the observed relationship between life-history traits and microevolutionary measures of selection can be skewed in empirical studies (Deinum et al. 2015; James and Eyre-Walker 2020), as the two measures show different sensitivity to a change in population size.

To capture changes in selection pressure following more recent population size fluctuations, our analytical results suggest to instead apply a combination of microevolutionary measures of selection and genetic drift. An example of such application is given by the comparison of island versus mainland populations where island colonization happened in the more recent past (James et al. 2016; Leroy et al. 2021). As microevolutionary measures of selection, not only $\left(\pi_{N}/\pi_{S}{)}^{\kappa }(t\right)$ but also polymorphism-based estimates of the DFE might be considered (Welch et al. 2008; Arunkumar et al. 2014; Chen et al. 2017). The choice of ${\bar{\omega }}^{\kappa }(t)$ for short branches, i.e. small *t*, as an alternative microevolutionary measure should, on the other hand, be avoided for two reasons. First, estimation of $\bar{\omega }$ has been shown to be significantly biased by polymorphisms if applied to short branches (Mugal et al. 2014, 2020), and therefore reflects the ongoing selection pressure in a population only poorly. In addition, the number of nonsynonymous and synonymous fixations is strongly influenced by the ancestral population size during a representative period of time after the change in population size (fig. 4*C* and *D*). These two reasons could, for example, explain the rather weak selection–drift relationship in the comparison of island and mainland species in some earlier studies that predate the re-sequencing era (Woolfit and Bromham 2005; Wright et al. 2009), and it could be interesting to reassess signatures of selection with help of polymorphism data.

Moreover, our analytical results entail important implications for the estimation of the rate of adaptive evolution, α. Many methods that estimate α, as for instance the DFE-alpha method and its derivatives (Keightley and Eyre-Walker 2007; Charlesworth and Eyre-Walker 2008; Eyre-Walker and Keightley 2009; Schneider et al. 2011; Kousathanas and Keightley 2013), are designed to contrast polymorphism-based and divergence-based data, i.e. they combine micro- and macroevolutionary measures. However, the different sensitivity in $\left(\pi_{N}/\pi_{S}{)}^{\kappa }(t\right)$ and ${\bar{\omega }}^{\kappa }(t)$ after a rather recent change in population size causes a smaller value of ${\bar{\omega }}^{\kappa }(t)$ than of $\left(\pi_{N}/\pi_{S}{)}^{\kappa }(t\right)$ for a nonnegligible time period in case of a decline in population size (figs. 2 and 5). In the case of an increase in population size, ${\bar{\omega }}^{\kappa }(t)$ exaggerates $\left(\pi_{N}/\pi_{S}{)}^{\kappa }(t\right)$ for a substantial amount of time, which could wrongly be attributed to the presence of positive selection. Applying the DFE-alpha method for the estimation of α to populations in nonequilibrium conditions can consequently lead to confounded estimates: negative estimates of α can be obtained in case of a more recent decline (Gossmann et al. 2010; Good et al. 2013; Deinum et al. 2015) or an inflated α for a rather recent growth (Tsagkogeorga et al. 2012; Lin et al. 2018; Rousselle et al. 2018). Even though the more recent versions of these methods account for a change in population size, they neither directly account for the nonequilibrium period, nor address the discrepancies that arise as a result of the different timescales evaluated. Similarly, also a recent method by Brevet and Lartillot (2021) combines micro- and macroevolutionary measures of selection to estimate $N_{\mathrm{eff}}$ without accounting for the possibility that different $N_{\mathrm{eff}}$ could act on different timescales. Again, this can result in biased estimates.

Nevertheless, it could still be of interest to investigate the selection–drift relationship of both micro- and macroevolutionary measures of $N_{\mathrm{eff}}$ and natural selection. In fact, valuable information can be gained by comparison. If the observed relationships show a different behavior, this could be indicative of an ongoing nonequilibrium condition (fig. 5). Obviously, data availability is an important prerequisite for such an analysis. Microevolutionary measures of genetic drift and natural selection can be directly computed based on population re-sequencing data as these measures rely on intra-species genomic variation. Also, a macroevolutionary measure of $N_{\mathrm{eff}}$ can be assessed based on intra-species genomic variation (Leroy et al. 2021) with help of methods based on the sequentially Markovian coalescent (SMC) (McKenna et al. 2010; Li and Durbin 2011; Schiffels and Durbin 2014). However, assessing macroevolutionary measures of selection can be complicated by the lack of a distantly related reference species (or lack of available genomic data thereof) (e.g. Muyle et al. 2020), which often is unavoidable in empirical studies.

### Limitations and Possible Extensions of the Model

We built our model of a nonequilibrium scenario on several simplifying assumptions of which one is the focus on a single change in population size. The advantage of such narrow focus is that interpretations are more straightforward. On the other hand, the framework we presented here is only directly comparable to a limited number of empirical scenarios, such as (but not limited to) reductions in effective population size due to isolation of island and mainland populations (Woolfit and Bromham 2005; Wang et al. 2014; Kutschera et al. 2020; Leroy et al. 2021). In addition, it might also be of interest to study periodic changes in population size, as for example done by Rousselle et al. (2018) using simulations, or stochastically varying population size, as for example in Sjödin et al. (2004). Our analytical modeling approach, especially the derivation of a nonequilibrium AFS in the Poisson random field framework, may be extended to such situations. Periodic changes in population size would entail a sequence $t_{k}^{*}=kT$, for k=0,1,…, of time points of period *T* and a sequence $\kappa_{0},\kappa_{1},\ldots$ of size parameters making up a new step function $N_{\kappa }$. For $t\in [t_{n-1}^{*},t_{n}^{*})$, the more general nonequilibrium AFS extending equation (6) is

$$\begin{array}{rl} \lim_{N\to \infty }E\langle X_{t}^{N_{\kappa }},f\rangle & =\theta \int_{0}^{1}f(y)\omega_{\gamma ,\kappa_{0}}\psi_{\gamma ,\kappa_{0}}(y)\;dy\\ & \;+\theta f(1)\int_{0}^{t}\sum_{k=1}^{n}\omega_{\gamma ,\kappa_{k-1}}1_{\left\{t_{k-1}^{*}<s\leq t_{k}^{*}\right\}}\;ds\\ & \;+\theta \sum_{k=1}^{n}\int_{0}^{1}[f(y)-E_{y}^{\gamma ,\kappa_{k-1}}f(\xi_{t_{k}^{*}\wedge t}^{t_{k-1}^{*}})]\\ & \;\times [\omega_{\gamma ,\kappa_{k}}\psi_{\gamma ,\kappa_{k}}(y)-\omega_{\gamma ,\kappa_{k-1}}\psi_{\gamma ,\kappa_{k-1}}(y)]\;dy. \end{array}$$

In fact, this AFS is not restricted to periodically changing environments, and hence may be used to develop the further case of allowing a prescribed, continuously varying, deterministic, scaled population size, such that N(t)/N→κ(t). To include the perspective of effective population size itself evolving as a stochastic process, consider the case where the population size is switching between the two states *N* and κN according to a continuous time Markov chain with given transition rates. A simulation study of the discrete time version of this model in the context of coalescent effective population size is carried out in Sjödin et al. (2004), and potential implications are discussed in terms of fast, intermediate, or slow fluctuations. Quite similar considerations might be relevant in the situation at hand.

Apart from change in population size, there are other mechanisms that can cause a demographic nonequilibrium and affect the selection–drift balance. Examples of such mechanisms are population structure or migration. Inference of estimates of gene flow in nonequilibrium conditions exemplify that migration can impact inference from genomic data (e.g. Austin et al. 2004; Pinho et al. 2008). It could therefore be interesting to extend the description of the nonequilibrium AFS and incorporate migration to investigate its effect on the selection–drift relationship.

Besides demography, also linkage among sites influences allele frequency trajectories. Interference of allele frequency trajectories among two selected sites results in a reduced efficacy of selection, a phenomenon known as Hill–Robertson effect (Hill and Robertson 1966). In addition, the effect of selection at linked sites affects allele frequency trajectories at neutral sites, which implies that neutral diversity is affected indirectly by selection (Maynard Smith and Haigh 1974; Charlesworth et al. 1993; Campos et al. 2014; Hollister et al. 2014). The phenomenon of selection at linked sites has recently received much attention. Specifically, a debate on the validity of the (nearly) neutral theory in the light of linked selection effects has originated (Jensen et al. 2018; Kern and Hahn 2018; Chen et al. 2020). Kern and Hahn (2018) triggered the debate that with today’s data and knowledge the theory lacks evidence as genomic variation is widely shaped by “the direct and indirect consequences of natural selection”. This prompted efforts to reconcile the original theory with new insights, which suggest that the nearly neutral theory does not lose its validity *per se* but rather that its initial formulation needs to be extended to account for selection at linked sites (Jensen et al. 2018; Chen et al. 2020).

Recently, Torres et al. (2018, 2020) and Johri et al. (2020) also discussed the interaction of a demographic nonequilibrium and selection at linked sites on allele frequency trajectories of neutral sites. Their simulation results provide evidence that the AFS at neutral sites provides a biased picture of the demographic history, since selection at linked sites shows a significant impact on the shape of the AFS. In addition, the authors highlight that conventional methods used to infer the DFE that do not account for linked selection, such as the widely used DFE-alpha method (Keightley and Eyre-Walker 2007; Eyre-Walker and Keightley 2009), provide biased estimates. To account for any effects of linkage among sites, Johri et al. (2020) propose an ABC approach to estimate the DFE. Essentially, the comparison between their ABC approach and conventional approaches stresses the importance of a refined null model that accounts for the interaction between demography and selection at linked sites, i.e. indirect selection.

Complementary to Johri et al. (2020), analytical results gained in the present study stress that besides the interaction of demography and indirect selection, also the interaction between a demographic nonequilibrium and direct selection is important. This suggests that observed differences between the DFE-alpha method (Keightley and Eyre-Walker 2007; Eyre-Walker and Keightley 2009) and the ABC method (Johri et al. 2020) should be attributed to both, direct and indirect effects of a demographic nonequilibrium on allele frequency trajectories. In order to decompose the two effects within our mathematical framework, we would need to incorporate linked selection in our model. As an approximation, selection at linked sites can be modeled as variation in effective population size across the genome (Robertson 1961; Charlesworth et al. 2009), which also could be implemented in our framework. For complementary methodological developments, existing methodology that accounts for the direct effects of a demographic nonequilibrium (Williamson et al. 2005; Boyko et al. 2008) could be extended in a similar fashion.

### Conclusion

The flexible framework we present in this study allows for various modifications and extensions. At the same time, restricting the model by specific simplifying assumptions enables us to derive exact analytical solutions, which found the basis for valuable conceptual understanding. We demonstrate that the selection–drift balance is substantially affected by a change in population size. Moreover, we illustrate that micro- and macroevolutionary measures of natural selection and genetic drift show a considerably different sensitivity to recent fluctuations in size. These analytical results, therefore, serve as a helpful tool for empirical studies to choose suitable measures for investigating the selection–drift relationship and to correctly interpret and compare resulting observations. Finally, the explicit modeling of a nonequilibrium condition and its effects on allele frequency trajectories extends the existing body of population genetics theory and constitutes a valuable foundation to refine methodology.

## Materials and Methods

### The Poisson Random Field Model During Nonequilibrium

To construct the random measure $X_{t}^{N_{\kappa }}$, briefly introduced in the “Basic Model”, we apply stochastic Poisson integrals. First, for $t\leq t^{*}$,

(7) $$\langle X_{t}^{N_{\kappa }},f\rangle =\int_{0}^{1}\int_{D}f(\xi_{t}^{0})M_{\gamma }(dy,d\xi^{0})+\int_{0}^{t}\int_{D}f(\xi_{t}^{s})N_{N_{\kappa }}(ds,d\xi^{s}),$$

for $f\in \tilde{F}$, specified in the Supplementary Section 1.1, Supplementary Material online, satisfying sufficient conditions for these integrals to be well defined. The class D is the path space for the diffusion processes $t\mapsto \xi_{t}$, consisting of functions g:R→[0,1] which are right continuous and have left limits. Moreover, $M_{\gamma }(dy,d\xi^{0})$ is a Poisson random measure on [0,1]×D with intensity $m_{\gamma ,1}(dy,d\xi^{0})=\theta \omega_{\gamma ,1}\psi_{\gamma ,1}(y)\;dy\;P_{y}^{\gamma ,1}(d\xi^{0})$ and $N_{N_{\kappa }}(ds,d\xi^{s})$ is a Poisson random measure on R×D with intensity measure $n_{N_{\kappa }}(ds,d\xi^{s})=\theta N_{\kappa }(s)\;ds\;P_{1/N_{\kappa }}^{\gamma ,N_{\kappa }/N}(d\xi^{s})$. The first term in equation (7) represents the family of ancestral allele frequencies with initial values at t=0 given by the Poisson measure $X_{0}^{N_{\kappa }}(dy)$, 0<y<1. The second term contains additional allele frequencies due to mutations during [0,t]. Similarly, at a time $t>t^{*}$ we have

(8) $$\langle X_{t}^{N_{\kappa }},f\rangle =\int_{0}^{1}\int_{D}f(\xi_{t}^{t^{*}})M_{\gamma ,\kappa }^{X_{t^{*}}}(dy,d\xi^{t^{*}})+\int_{t^{*}}^{t}\int_{D}f(\xi_{t}^{s})N_{N_{\kappa }}(ds,d\xi^{s}).$$

Conditional on $X_{t^{*}}$, $M_{\gamma ,\kappa }^{X_{t^{*}}}(dy,d\xi^{t^{*}})$ is a Poisson random measure on (0,1]×D with intensity $X_{t^{*}}^{N_{\kappa }}(dy)\;P_{y}^{\gamma ,\kappa }(d\xi^{t^{*}})$. This term represents the fate of the allele frequencies extending beyond $t^{*}$ of all alleles, polymorphic or fixed, which were present at $t^{*}$. The second term covers mutations occurring in $\left(t^{*},t\right)$.

To analyze the nonequilibrium AFS caused by applying population size $N_{\kappa }$, we derive the limiting expected value of $X_{t}$, see Theorem 1 in Supplementary Section 1.2, Supplementary Material online. The ancestral component, mutations which occurred prior to $t^{*}$, yields

(9) $$\begin{array}{rl} & \lim_{N\to \infty }E\left[\int_{0}^{1}\int_{D}f(\xi_{t}^{t^{*}})M_{\gamma ,\kappa }^{X_{t^{*}}}(dy,d\xi^{t^{*}})\right]\\ & \;=\theta \omega_{\gamma ,1}f(1)t^{*}+\theta \int_{0}^{1}E_{y}^{\gamma ,\kappa }[f(\xi_{t}^{t^{*}})]\omega_{\gamma ,1}\psi_{\gamma ,1}(y)\;dy, \end{array}$$

compare equation (IV) in Theorem 1. Similarly, the allele frequencies originating from mutations starting at $t^{*}$ generate the nonstationary build-up AFS (Kaj and Mugal 2016, Theorem 1), that arises from a completely mono-allelic population,

(10) $$\begin{array}{rl} & \lim_{N\to \infty }E\left[\int_{t^{*}}^{t}\int_{D}f(\xi_{t}^{s})\;N_{N_{\kappa }}(ds,d\xi^{s})\right]=\theta \;\omega_{\gamma ,\kappa }f(1)(t-t^{*})\\ & \;+\theta \int_{0}^{1}\left\{f(y)-E_{y}^{\gamma ,\kappa }[f(\xi_{t}^{t^{*}})]\right\}\omega_{\gamma ,\kappa }\psi_{\gamma ,\kappa }(y)\;dy. \end{array}$$

### The Ratio of Nucleotide Diversity During Nonequilibrium

We derive and investigate the ratio of nucleotide diversity, $\pi_{N}/\pi_{S}$ (Nei and Li 1979), in a population undergoing a change in population size according to $N_{\kappa }$. Nucleotide diversity measures the number of pairwise differences, which entails integrating the specific function $f_{\mathrm{pw}}(y)=2y(1-y)$, the probability of sampling pairwise differences at frequency *y*, with respect to the AFS. As a reference case we observe that during equilibrium in a population controlled by a size parameter κ and a fixed selection coefficient γ≠0, we have by equation (5) with $f_{\mathrm{pw}}(1)=0$,

(11) $$\pi_{N}^{\gamma ,\kappa }=\theta \int_{0}^{1}2y(1-y)\omega_{\gamma ,\kappa }\psi_{\gamma ,\kappa }(y)\;dy=4\theta \kappa \left(\frac{1}{1-e^{-2\gamma \kappa }}-\frac{1}{2\gamma \kappa }\right).$$

More generally, by applying equation (6), we obtain the time-dependent nonsynonymous nucleotide diversity measure $\pi_{N}^{\gamma ,\kappa }(t)=\lim_{N\to \infty }E\langle X_{t}^{N_{\kappa }},f_{\mathrm{pw}}\rangle$ in nonequilibrium.

In order to allow for variation in selection across sites for the nonsynonymous diversity, we integrate the previous expressions over a DFE. We denote the random variable generating the values for γ by V and assume it has a continuous density function $h_{V}$. Because of the presumed rarity or negligibility of advantageous mutations within the nearly neutral theory, we focus on weak and strong purifying selection following Eyre-Walker et al. (2006), Loewe and Charlesworth (2006) and Galtier and Rousselle (2020). A common choice of DFE in this scenario is the negative Γ-distribution. The density function is

(12) $$h_{V}(v)=\frac{(-v{)}^{a-1}\;e^{v/b}}{b^{a}\Gamma (a)},\;v\leq 0,$$

with shape parameter a>0, scale parameter b>0, and mean −ab. Integration of the expression in equation (11) and $\pi_{N}^{\gamma ,\kappa }(t)$ over this density yields an averaged diversity measure $\pi_{N}^{\kappa }$. Taken together it holds

(13) $$\pi_{N}^{\kappa }(t)=\left\{ \begin{array}{ll} E\pi_{N}^{V,1}, & t\leq t^{*},\\ E\pi_{N}^{V,\kappa }+2\theta E\int_{0}^{1}E_{y}^{V,\kappa }[\xi_{t}^{t^{*}}(1-\xi_{t}^{t^{*}})]\; & \\ \;\times [\omega_{V,1}\psi_{V,1}(y)-\omega_{V,\kappa }\psi_{V,\kappa }(y)]\;dy, & t>t^{*}, \end{array}\right.$$

see Supplementary Section 1.3, Supplementary Material online for details. The expectations in the above expression are used to indicate integration over the DFE. We observe that $\pi_{N}^{\kappa }(t)$ approaches a new equilibrium, $\pi_{N}^{\kappa }(t)\to E\pi_{N}^{V,\kappa }$ as t→∞, since $\xi_{t}^{t^{*}}\in \{0,1\}$ for t→∞. For the case of neutral evolution, γ=0, the result simplifies considerably and we obtain the synonymous diversity as

(14) $$\pi_{S}^{\kappa }(t)=\left\{ \begin{array}{ll} 2\theta , & t\leq t^{*},\\ 2\theta \kappa +2\theta (1-\kappa )\;e^{-(t-t^{*})/\kappa }, & t>t^{*}, \end{array}\right.$$

with $\pi_{S}^{\kappa }(t)\to 2\theta \kappa$ as t→∞. The ratio of nonsynonymous and synonymous diversity, which we denote $\left(\pi_{N}/\pi_{S}{)}^{\kappa }(t)\;:=\pi_{N}^{\kappa }(t)/\pi_{S}^{\kappa }(t\right)$, is determined by equations (13) and (14).

### The Ratio of Nonsynonymous and Synonymous Fixations

We consider the number of nonsynonymous and synonymous fixations (Goldman and Yang 1994; Muse and Gaut 1994) in the population during the finite time interval [0,t] with a change in population size as before given by $N_{\kappa }$. To account for fixations in the random field setting, we wish to count all Poisson points $\left(s,\xi^{s}\right)$ such that $\xi_{t}^{s}=1$. In other words, we evaluate the indicator function $f_{\mathrm{fix}}(y)\;:=1_{\left\{1\right\}}(y)$ at the nonequilibrium AFS, equation (6). Hence, the number of fixations in the population during [0,t] with a change in population size given by $N_{\kappa }$ is $Z^{\gamma ,\kappa }(t)\;:=\lim_{N\to \infty }E\langle X_{t}^{N_{\kappa }},f_{\mathrm{fix}}\rangle$. The decomposition of $\lim_{N\to \infty }E\langle X_{t}^{N_{\kappa }},f_{\mathrm{fix}}\rangle$ into the ancestral contribution in equation (9) and the build-up in equation (10) allows for matching the different categories of fixations in figure 1*A* with the corresponding analytic representation: the first term in equation (9) reflects fixations (blue paths) appearing during $\left[0,t^{*}\right]$, whereas the second part corresponds to fixations (blue-red paths) during $\left[t^{*},t\right]$ for which the mutation happened before $t^{*}$. The build-up component in equation (10) accounts for fixations (red paths) during $\left[t^{*},t\right]$ for which the mutation occurred after $t^{*}$.

To obtain an explicit representation of $Z^{\gamma ,\kappa }(t)$, we note that $f_{\mathrm{fix}}(1)=1$ and that the expectation operator applied to $f_{\mathrm{fix}}(y)$ can be rewritten in terms of the fixation time distribution,

(15) $$E_{y}^{\gamma ,\kappa }[f_{\mathrm{fix}}(\xi_{t})]=E_{y}^{\gamma ,\kappa }[1_{\left\{1\right\}}(\xi_{t})]=P_{y}^{\gamma ,\kappa }(\xi_{t}=1)=P_{y}^{\gamma ,\kappa }(\tau_{1}\leq t).$$

Thus,

$$Z^{\gamma ,\kappa }(t)=\left\{ \begin{array}{ll} \theta \omega_{\gamma ,1}t, & t\leq t^{*},\\ \theta \{\omega_{\gamma ,1}t^{*}+\omega_{\gamma ,\kappa }(t-t^{*})\} & \\ \;\;+\theta \int_{0}^{1}P_{y}^{\gamma ,\kappa }(\tau_{1}\leq t-t^{*})\\ \;\;\times [\omega_{\gamma ,1}\psi_{\gamma ,1}(y)-\omega_{\gamma ,\kappa }\psi_{\gamma ,\kappa }(y)]\;dy, & t>t^{*}, \end{array}\right.$$

compare Supplementary Section 1.4, Supplementary Material online for technical details. Fixations that originate from nonsynonymous mutations are averaged over the DFE in equation (12); for synonymous fixations γ is set to zero. Finally, the ratio of nonsynonymous and synonymous fixations after a change in population size is defined as

$${\bar{\omega }}^{\kappa }(t)=\frac{EZ^{V,\kappa }(t)}{Z^{0,\kappa }(t)}.$$

The nonequilibrium quantity ${\bar{\omega }}^{\kappa }(t)$ is consistent with the equilibrium, instantaneous fixation rate ratio $\omega_{\gamma ,\kappa }$ stated in equation (3), since ${\bar{\omega }}^{\kappa }(t)=E\omega_{V,1}$ for $t\leq t^{*}$ and ${\bar{\omega }}^{\kappa }(t)\to E\omega_{V,\kappa }$ for t→∞.

### Stochastic Simulations

For performing stochastic simulation of paths $(\xi_{t}{)}_{t}$ in the Julia programming language (Bezanson et al. 2017), we apply the discrete Wright–Fisher model with selection to a population of size *N*. It suffices to simulate paths for the reference population, since a polymorphism in a population of size κN evolves as in the reference population but with time scaled by κ. Paths are simulated over a maximum of *n* generations using the binomial Wright–Fisher sampling with selection. The selection coefficient in the discrete setting is obtained from the relation s=γ/N.

For the distribution of the time to fixation, $P_{y}^{\gamma ,\kappa }(\tau_{1}\leq t)$, we generate $m_{\tau_{1}}$ paths for each tuple (y,γ). If the derived allele does not get fixed, the time to fixation is set to infinity. Otherwise the fixation time is set to the generation it got fixed. Finally, the distribution function of the time to fixation on the evolutionary timescale is obtained by scaling generations with *N*. For the expected value $E_{y}^{\gamma ,\kappa }[f(\xi_{t}^{t^{*}})]$ in the nonequilibrium AFS we simulate and average over $m_{\xi }$ paths for each triplet $\left(y,\gamma ,(t-t^{*})/\kappa \right)$.

Parameters used are N=1000, n=20000, s∈[−1,0] or equivalently γ∈[−1000,0], $m_{\tau_{1}}=100\;000$ (if γ=0) and $m_{\tau_{1}}=10\;000$ (if γ<0), respectively, $m_{\xi }=1\;000$.

## Supplementary Material

Supplementary data are available at *Genome Biology and Evolution online*.

## Supplementary Material

evac058_Supplementary_Data

## Data Availability

Code used to implement the stochastic simulations can be found on GitLab.
