# Supplementary material for: A Nearly Neutral Model of Molecular Signatures of Natural Selection after Change in Population Size
Source: Genome Biol Evol. 2022 Apr 27;14(5):evac058. doi: 10.1093/gbe/evac058 (PMC9127441; doi:10.1093/gbe/evac058)
Supplement: evac058_Supplementary_Data [file evac058_supplementary_data.zip › supplement.pdf]

Supplemental Material

A Nearly-Neutral Model of Molecular Signatures of Natural Selection after Change in Population Size

Rebekka Müller, Ingemar Kaj, Carina F. Mugal*

Carina F. Mugal: Carina.Mugal@ebc.uu.se

Table of Contents

1 Supplemental Analytical Derivations **2**

1.1 The AFS for the equilibrium case 2

1.2 The nonequilibrium AFS after a change in population size 9

1.3 Nucleotide diversity 11

1.4 Number of fixations 12

2 Supplemental Figures **14**

1. Supplemental Analytical Derivations

1.1. The AFS for the equilibrium case

We begin with the reference case $\kappa = 1$ of fixed population size and consider the Wright-Fisher diffusion $\xi^s = (\xi^s_t)_{t \geq s}$, $\xi^s_s = y$, with law $\mathbb{P}^\gamma_y(d\xi^s)$. To emphasize the initial value we may write $(\xi^{s,y}_t)_{t\geq s}$. The associated expectation operator $T^\gamma_{t-s}f(y) = \mathbb{E}^\gamma_y[f(\xi^s_t)], t \geq s$, satisfies the semigroup property $T^\gamma_s(T^\gamma_{t-s}f)(y) = T_t f(y)$, and the diffusion infinitesimal generator is the differential operator

$$Q_\gamma f(x) = \gamma x(1-x)\, f'(x) + \frac{1}{2}x(1-x)\, f''(x), \quad f \in \mathcal{D},$$

for a suitable domain $\mathcal{D}$ of twice differentiable functions on the unit interval. We denote by $\mathcal{F}$ the class of real-valued bounded functions on $[0, 1]$ with $f(0) = 0$, satisfying $\int y^{-1}|f(y)|\, dy < \infty$, and put

$$\widetilde{\mathcal{F}} = \{f \in \mathcal{F} \cap \mathcal{D} : Q_\gamma f \in \mathcal{F}\}.$$

Similarly, $\mathcal{F}_0$ and $\widetilde{\mathcal{F}}_0$ denote the further restricted classes of functions which, in addition, satisfy $f(1) = 0$. It is convenient to derive the following property first for the restricted class of functions vanishing at both boundary points, case i), and then make the necessary observations for handling the larger class of functions, as case ii).

Lemma 1. *For each $t \geq 0$,*

i) *if $f \in \widetilde{\mathcal{F}}_0$ then $T^\gamma_t f \in \mathcal{F}_0$; and*

ii) *if $f \in \widetilde{\mathcal{F}}$ then $T^\gamma_t f \in \mathcal{F}$.*

Proof. For statement i) let $f \in \widetilde{\mathcal{F}}_0$. Clearly, $T^\gamma_t f$ is bounded with $T^\gamma_t f(0) = T^\gamma_t f(1) = 0$. We need to show $\int_0^1 x^{-1}|T^\gamma_t f(x)|\, dx < \infty$.

As a consequence of Itô's formula we have the basic relation

$$T_t^\gamma f(x) = f(x) + \mathbb{E}_x^\gamma\Big[\int_0^t Q_\gamma f(\xi_r)\,dr\Big],$$

and so

$$|T_t^\gamma f(x) - f(x)| \le \mathbb{E}_x^\gamma\Big[\int_0^t |Q_\gamma f(\xi_r)|\,dr\Big].$$

Let τ be the absorption time of (ξ_t). Then, since $Q_\gamma f \in \mathcal{F}_0$ by assumption,

$$\mathbb{E}_x^\gamma\Big[\int_0^t |Q_\gamma f(\xi_r)|\,dr\Big] = \mathbb{E}_x^\gamma\Big[\int_0^{t\wedge\tau} |Q_\gamma f(\xi_r)|\,dr\Big] \le \mathbb{E}_x^\gamma\Big[\int_0^{\tau} |Q_\gamma f(\xi_r)|\,dr\Big]$$

so that

$$|T_t^\gamma f(x) - f(x)| \le \mathbb{E}_x^\gamma\Big[\int_0^{\tau} |Q_\gamma f(\xi_r)|\,dr\Big] = \int_0^1 |Q_\gamma f(y)| G_\gamma(x,y)\,dy,$$

where $G_\gamma(x,y)$ is the Green function of the Wright-Fisher diffusion. Hence,

$$\begin{aligned}\int_0^1 x^{-1}|T_t^\gamma f(x) - f(x)|\,dx &\le \int_0^1\int_0^1 |Q_\gamma f(y)| x^{-1} G_\gamma(x,y)\,dydx \\ &\le \sup_{x,y} |x^{-1}G_\gamma(x,y)y| \int_0^1 y^{-1}|Q_\gamma f(y)|\,dy\end{aligned}$$

and therefore, uniformly in t,

$$\begin{aligned}\int_0^1 x^{-1}|T_t^\gamma f(x)|\,dx \le& \int_0^1 x^{-1}|f(x)|\,dx \\ &+ \sup_{x,y} |x^{-1}G_\gamma(x,y)y| \int_0^1 y^{-1}|Q_\gamma f(y)|\,dy.\end{aligned} \tag{I}$$

As the two integrals on the right hand side are finite by assumption, it remains to show that

$$x^{-1}G_\gamma(x,y)y = \mathbb{1}_{\{x<y\}} x^{-1} q_\gamma(x)\psi_\gamma(y)y + \mathbb{1}_{\{x>y\}} x^{-1}(1-q_\gamma(x))\tilde{\psi}_\gamma(y)y$$

is bounded on the unit square $0 \le x, y \le 1$. Here, $q_\gamma(x)$ is defined in Eq. (2), $\psi_\gamma(y)$ in Eq. (4), and

$$\tilde{\psi}_\gamma(y) = \frac{e^{2\gamma y} - 1}{\gamma y(1-y)}, \quad \gamma \ne 0, \qquad \tilde{\psi}_0(y) = \frac{2}{1-y}.$$

For $\gamma = 0$,

$$x^{-1}G_0(x,y)y = \mathbb{1}_{\{x<y\}}2 + \mathbb{1}_{\{x>y\}}\frac{1-x}{x}\frac{2y}{1-y} \le 2.$$

For $\gamma \neq 0$, the nonnegative function $x^{-1}G_\gamma(x,y)y$ has the representation and upper bound

$$\begin{aligned}
&\frac{4\gamma}{1-e^{-2\gamma}}\Big\{\mathbb{1}_{\{x<y\}}\frac{1-e^{-2\gamma x}}{2\gamma x}\frac{1-e^{-2\gamma(1-y)}}{2\gamma(1-y)} + \mathbb{1}_{\{x>y\}}\frac{e^{-2\gamma x}-e^{-2\gamma}}{2\gamma x}\frac{e^{2\gamma y}-1}{2\gamma(1-y)}\Big\} \\
&\le \frac{4\gamma}{1-e^{-2\gamma}}\Big\{\mathbb{1}_{\{x<y\}}\frac{1-e^{-2\gamma x}}{2\gamma x}\frac{1-e^{-2\gamma(1-y)}}{2\gamma(1-y)} + \mathbb{1}_{\{x>y\}}\frac{e^{-2\gamma x}-e^{-2\gamma}}{2\gamma(1-x)}\frac{e^{2\gamma y}-1}{2\gamma y}\Big\}.
\end{aligned}$$

From here using straightforward estimates one obtains, for example,

$$\sup_{0\le x,y\le 1} x^{-1}G_\gamma(x,y)y \le \frac{e^{2|\gamma|}-1}{|\gamma|},$$

which concludes the proof that $T_t^\gamma f$ belongs to $\mathcal{F}_0$.

Turning to statement ii), by using Kaj and Mugal (2016, Lemma 1, Eq. (30)), we now have

$$T_t^\gamma f(x) = f(x) + \mathbb{E}_x^\gamma\Big[\int_0^{t\wedge\tau}(Q_\gamma f(\xi_r) - Q_\gamma f(1)q_\gamma(\xi_r))\,dr\Big] + Q_\gamma f(1)q_\gamma(x)\,t.$$

Since $q_\gamma(1) = 1$, the integrand function $y \mapsto Q_\gamma f(y) - Q_\gamma f(1)q_\gamma(y)$ belongs to $\mathcal{F}_0$. Hence we can proceed as above and obtain as replacement of Eq. (I),

$$\begin{aligned}
\int_0^1 x^{-1}|T_t^\gamma f(x)|\,dx &\le \int_0^1 x^{-1}|f(x)|\,dx + Q_\gamma f(1)\,t\int_0^1 x^{-1}q_\gamma(x)\,dx \\
&\quad + \sup_{x,y}|x^{-1}G_\gamma(x,y)y|\int_0^1 y^{-1}|Q_\gamma f(y) - Q_\gamma f(1)q_\gamma(y)|\,dy,
\end{aligned}$$

where the supremum is the same factor already treated under i). The observation

$$\int_0^1 x^{-1}q_\gamma(x)\,dx < \infty,$$

completes the proof. $\square$

The stationary AFS arises in the Poisson random field approach as a limiting intensity measure of a random Poisson measure which adds up the allele contributions from all mutations in the infinite past. Formally, let $\mathcal{N}_N(ds, dy, d\xi^s)$ be a Poisson measure on $\mathbb{R} \times [0,1] \times \mathcal{D}$ with intensity $N ds \delta_{1/N}(dy) \mathbb{P}^\gamma_y(d\xi^s)$, and let $\mathcal{N}_\gamma(dy)$ be a Poisson random measure on $[0,1]$ with intensity measure $\omega_\gamma \psi_\gamma(y) dy$, where ω_γ is the scaled fixation probability introduced in Eq. (3). Note that for simplicity we assume $\theta = 1$ throughout Section 1.1 and Section 1.2 of the supplemental material. For $f \in \mathcal{F}_0$, we have for fixed t as $N \to \infty$ the convergence in distribution

$$\int_{-\infty}^{t} \int \int_{\mathcal{D}} f(\xi_t^{s,y})\, \mathcal{N}_N(ds, dy, d\xi^{s,y}) \Rightarrow \int_0^1 f(y)\, \mathcal{N}_\gamma(dy)$$

and the associated convergence of expected values

$$\mathbb{E}\Big[\int_{-\infty}^{t} \int \int_{\mathcal{D}} f(\xi_t^{s,y})\, \mathcal{N}_N(ds, dy, d\xi^{s,y})\Big] \to \omega_\gamma \int_0^1 f(y) \psi_\gamma(y)\, dy$$

(Kaj and Mugal 2016, Section 2.4.). Each Poisson point in $\mathcal{N}_N$ represents the allele frequency of a mutation starting at time s at a fraction $1/N$ of the population. Taking all mutations with $s \leq t$ together and considering the resulting spectrum of polymorphic sites at t, we obtain $\mathcal{N}_\gamma$.

Next, we decompose the stationary Poisson measure $\mathcal{N}_\gamma$ at a fixed time $t \geq 0$, in two independent contributions by splitting up the mutation times, separating $s \leq 0$ and $0 < s \leq t$, so that

$$\mathbb{E}\Big[\Big\{ \int_{-\infty}^{0} + \int_0^t \Big\} \int \int_{\mathcal{D}} f(\xi_t^{s,y})\, \mathcal{N}_N(ds, dy, d\xi^{s,y})\Big] \to \omega_\gamma \int_0^1 f(y) \psi_\gamma(y)\, dy. \qquad \text{(II)}$$

The limit of the first term in the decomposition is obtained in the following Lemma 2, relation ii). The limit of the second term follows immediately, relation iii). Let $\mathcal{M}_\gamma(dy, d\xi^{0,y})$ be

a Poisson random measure on $[0,1]\times\mathcal{D}$ with intensity $m_\gamma(dy,d\xi^{0,y}) = \omega_\gamma\psi_\gamma(y)dy\,\mathbb{P}_y^\gamma(d\xi^0)$. The Poisson points $(y,\xi^{0,y})$ are paths with initial state $\xi_0 = y$ according to the stationary intensity $\omega_\gamma\psi_\gamma(y)dy$ and evolve as Wright-Fisher diffusions $(\xi_t^{0,y})_{t\geq 0}$.

Lemma 2. *For $f \in \widetilde{\mathcal{F}}_0$ we have as $N\to\infty$ the convergence in distribution,*

$$\text{i)}\quad \int_{-\infty}^0\int\int_{\mathcal{D}} f(\xi_t^{s,y})\,\mathcal{N}_N(ds,dy,d\xi^{s,y}) \Rightarrow \int_{[0,1]\times\mathcal{D}} f(\xi_t^{0,y})\,\mathcal{M}_\gamma(dy,d\xi^{0,y})$$

and convergence of expected values,

$$\text{ii)}\quad \mathbb{E}\Big[\int_{-\infty}^0\int\int_{\mathcal{D}} f(\xi_t^{s,y})\,\mathcal{N}_N(ds,dy,d\xi^{s,y})\Big] \to \int_0^1 T_t^\gamma f(y)\omega_\gamma\psi_\gamma(y)\,dy,$$

and

$$\text{iii)}\quad \mathbb{E}\Big[\int_0^t\int\int_{\mathcal{D}} f(\xi_t^{s,y})\,\mathcal{N}_N(ds,dy,d\xi^{s,y})\Big] \to \int_0^1 (f(y) - T_t^\gamma f(y))\omega_\gamma\psi_\gamma(y)\,dy.$$

Proof. The logarithmic moment generating functional of the N-dependent Poisson integral equals

$$\ln\mathbb{E}\Big[\exp\Big\{\int_{-\infty}^0\int\int_{\mathcal{D}} f(\xi_t^{s,y})\mathcal{N}_N(ds,dy,d\xi^{s,y})\Big\}\Big] = N\int_{-\infty}^0 \mathbb{E}_{1/N}^\gamma[e^{f(\xi_t^s)}-1]\,ds. \tag{III}$$

Here, conditioning on $\{\xi_0^s\}_{s<0}$,

$$\begin{aligned}\int_{-\infty}^0 \mathbb{E}_x^\gamma[e^{f(\xi_t^s)}-1]\,ds &= \int_{-\infty}^0 \mathbb{E}_x^\gamma\mathbb{E}_{\xi_0^s}^\gamma[e^{f(\xi_t^0)}-1]\,ds\\ &= \mathbb{E}_x^\gamma\Big[\int_{-\infty}^0 T_t(e^f-1)(\xi_0^s)\,ds\Big] = \mathbb{E}_x^\gamma\Big[\int_0^\infty T_t(e^f-1)(\xi_r)\,dr\Big].\end{aligned}$$

Since $f\in\widetilde{\mathcal{F}}_0$ we have $e^f-1\in\widetilde{\mathcal{F}}_0$. By Lemma 1 i), $T_t(e^f-1)\in\mathcal{F}_0$, $t\geq 0$. Thus, the rightmost expression in Eq. (III) equals

$$N\mathbb{E}_{1/N}^\gamma\Big[\int_0^\tau T_t^\gamma(e^f-1)(\xi_r)\,dr\Big] = N\int_0^1 G_\gamma(1/N,y)T_t^\gamma(e^f-1)(y)\,dy.$$

Therefore, as $N \to \infty$,

$$N \int_{-\infty}^{0} \mathbb{E}^{\gamma}_{1/N}[e^{f(\xi^s_t)} - 1]\, ds \to \int_0^1 T^{\gamma}_t (e^f - 1)(y)\, \omega_\gamma \psi_\gamma(y)\, dy$$
$$= \ln \mathbb{E}\Big[\exp\Big\{ \int_{[0,1]\times\mathcal{D}} f(\xi^{0,y}_t)\, \mathcal{M}_\gamma(dy, d\xi^{0,y}) \Big\}\Big],$$

which shows convergence of the marginal distributions, for a fixed t. The convergence of the finite-dimensional distributions can be established as in Kaj and Mugal (2016, Proposition 2). Statement ii), which is

$$N \int_{-\infty}^{0} \mathbb{E}^{\gamma}_{1/N}[f(\xi^s_t)]\, ds \to \int_0^1 T^{\gamma}_t f(y)\, \omega_\gamma \psi_\gamma(y)\, dy,$$

follows as above, now using that $T^{\gamma}_t f \in \mathcal{F}_0$. Then we obtain iii) by subtracting ii) from Eq. (II). □

In view of Lemma 2 i), from now on we consider the allele frequency model given by the Poisson integral $\langle \mathcal{X}^N_t, f\rangle$, $t \geq 0$, defined by

$$\langle \mathcal{X}^N_t, f\rangle = \int_{[0,1]\times\mathcal{D}} f(\xi^{0,y}_t)\, \mathcal{M}_\gamma(dy, d\xi^{0,y}) + \int_0^t \int \int_{\mathcal{D}} f(\xi^{s,y}_t)\, \mathcal{N}_N(ds, dy, d\xi^{s,y}).$$

Here, the site frequency paths enter the system either at $t = 0$ according to a Poisson measure with intensity $\omega_\gamma \psi_\gamma(y)\, dy$, $0 \leq y \leq 1$, or at constant rate $N\, ds$, $s \geq 0$, with initial frequency $1/N$. In particular,

$$\langle \mathcal{X}^N_0, f\rangle = \int_0^1 f(y)\, \mathcal{N}_\gamma(dy)$$

is independent of N and we write $\mathcal{X}_0$. This setting allows us to analyze the effect of allele fixations, by dropping the restriction $f(1) = 0$ required for $\widetilde{\mathcal{F}}_0$. The next result shows that fixations build up at an asymptotically linear rate over time with the deviation from linearity controlled by

$$T^{\gamma}_t f(x) = \mathbb{P}^{\gamma}_x(\tau_1 \leq t) f(1) + \mathbb{E}^{\gamma}_x[f(\xi_t), \tau > t] \to \frac{1 - e^{-2\gamma x}}{1 - e^{-2\gamma}}\, f(1), \quad t \to \infty,$$

where τ_1 is fixation time and τ absorption time.

Lemma 3. *For each $f \in \widetilde{\mathcal{F}}$ as $N \to \infty$*

$$\text{i)}\quad \mathbb{E}\Big[\int_0^t \int \int_{\mathcal{D}} f(\xi_t^{s,y})\, \mathcal{N}_N(ds, dy, d\xi^{s,y})\Big] \\ \to \int_0^1 (f(y) - T_t^\gamma f(y))\, \omega_\gamma \psi_\gamma(y)\, dy + f(1)\, \omega_\gamma\, t,$$

$$\text{ii)}\quad \mathbb{E}_{\mathcal{X}_0}\langle \mathcal{X}_t^N, f\rangle \to \langle \mathcal{X}_0, T_t^\gamma f\rangle + \int_0^1 (f(y) - T_t^\gamma f(y))\, \omega_\gamma \psi_\gamma(y)\, dy + f(1)\, \omega_\gamma\, t,$$

$$\text{iii)}\quad \mathbb{E}\langle \mathcal{X}_t^N, f\rangle \to \int_0^1 f(y)\, \omega_\gamma \psi_\gamma(y)\, dy + f(1)\, \omega_\gamma\, t.$$

Proof. We begin with the basic representation of the expected value given by

$$\mathbb{E}\Big[\int_0^t \int \int_{\mathcal{D}} f(\xi_t^{s,y})\, \mathcal{N}_N(ds, dy, d\xi^{s,y})\Big] \\ = N\mathbb{E}_{1/N}^\gamma\Big[\int_0^t f(\xi_t^s)\, ds\Big] = N\mathbb{E}_{1/N}^\gamma\Big[\int_0^t f(\xi_u^0)\, du\Big].$$

and apply the rewriting

$$N\mathbb{E}_{1/N}^\gamma\Big[\int_0^t f(\xi_u)\, du\Big] = N\mathbb{E}_{1/N}^\gamma\Big[\int_0^{t\wedge\tau} (f(\xi_u) - f(1) q_\gamma(\xi_u))\, du\Big] \\ + f(1)\, N q_\gamma(1/N)\, t,$$

see Kaj and Mugal (2016, Section 6.3, Eq. (30)). Here, $N q_\gamma(1/N) \to \omega_\gamma$, $N \to \infty$. Moreover, the function g defined by $g(y) = f(y) - f(1) q_\gamma(y)$ belongs to $\widetilde{\mathcal{F}}_0$ and hence, by iii) in Lemma 2,

$$N\mathbb{E}_{1/N}^\gamma\Big[\int_0^{t\wedge\tau} g(\xi_u)\, du\Big] = N\mathbb{E}_{1/N}^\gamma\Big[\int_0^t g(\xi_u)\, du\Big] \\ \to \int_0^1 (g(y) - T_t^\gamma g(y)) \omega_\gamma \psi_\gamma(y)\, dy,$$

where

$$g(y) - T_t^\gamma g(y) = f(y) - T_t^\gamma f(y) + f(1)(T_t^\gamma q_\gamma(y) - q_\gamma(y)).$$

It is a well-known property of the Wright-Fisher diffusion, derived using Itô's formula, that $q_\gamma(\xi_t)$ is a P_x^γ-martingale, in particular $E_y^\gamma[q_\gamma(\xi_t)] = q_\gamma(y)$. Thus, $g(y) - T_t^\gamma g(y) = f(y) - T_t^\gamma f(y)$, and so

$$N\mathbb{E}_{1/N}^\gamma\Big[\int_0^t f(\xi_u)\,du\Big] \to \int_0^1 (f(y) - T_t^\gamma f(y))\omega_\gamma\psi_\gamma(y)\,dy + f(1)\,\omega_\gamma\,t,$$

as required to show i). Statements ii) and iii) follow by observing

$$\mathbb{E}_{\mathcal{X}_0}\Big[\int_{[0,1]\times\mathcal{D}} f(\xi_t^{0,y})\,\mathcal{M}_\gamma(dy, d\xi^{0,y})\Big] = \int_0^1 T_t^\gamma f(y)\,\mathcal{N}_\gamma(dy) = \langle \mathcal{X}_0, T_t^\gamma f\rangle$$

and

$$\mathbb{E}\int_0^1 T_t^\gamma f(y)\,\mathcal{N}_\gamma(dy) = \int_0^1 T_t^\gamma f(y)\omega_\gamma\psi_\gamma(y)\,dy.$$

□

1.2. The nonequilibrium AFS after a change in population size

The results in Section 1.1 extend to the (γ, κ) Wright-Fisher model with infinitesimal generator

$$Q_{\gamma,\kappa} f(x) = \gamma x(1-x)\,f'(x) + \frac{1}{2\kappa} x(1-x)\,f''(x), \quad f \in \mathcal{D},$$

by invoking the associated expectation operator $T_t^{\gamma,\kappa} f(x) = \mathbb{E}_x^{\gamma,\kappa}[f(\xi_t)]$, the scaled fixation probability $\omega_{\gamma,\kappa}$, and the intensity function $\psi_{\gamma,\kappa}(y)$, introduced in Eqs. (3) and (4). We are then in position to derive the AFS during nonequilibrium enforced by population size N_κ, that is, we run the system with $\kappa = 1$ up to a time point $t^* \geq 0$ and apply $\kappa \neq 1$ from thereon. The modified Poisson random measure, denoted $\mathcal{N}_{N_\kappa}(ds, dy, d\xi^{s,y})$, applies Poisson points for which the dynamics of the paths $(\xi_t^s)_{t\geq s}$ change with the current size of the population.

Theorem 1. *For $f \in \widetilde{\mathcal{F}}$ the nonequilibrium AFS at time $t \geq t^*$ after a change in population size at t^* is given by*

$$\lim_{N\to\infty} \mathbb{E}\,\langle \mathcal{X}_t^{N_\kappa}, f\rangle = f(1)\{\omega_{\gamma,1}\, t^* + \omega_{\gamma,\kappa}\,(t-t^*)\}$$
$$+\int_0^1 T_{t-t^*}^{\gamma,\kappa} f(y)(\omega_{\gamma,1}\psi_{\gamma,1}(y) - \omega_{\gamma,\kappa}\psi_{\gamma,\kappa}(y))\,dy + \int_0^1 f(y)\omega_{\gamma,\kappa}\psi_{\gamma,\kappa}(y)\,dy.$$

Proof. The allele frequencies originating from mutations before t^* form a stationary AFS with Poisson intensity $\omega_{\gamma,1}\psi_{\gamma,1}(y)\,dy$ at time t^*, compare Lemma 2 i) for $t = 0$. Hence,

$$\int_{-\infty}^{t^*}\int\int_{\mathcal{D}} f(\xi_{t^*}^{s,y})\,\mathcal{N}_{N_\kappa}(ds, dy, d\xi^{s,y}) \Rightarrow \int_0^1 f(y)\,\mathcal{N}_\gamma(dy).$$

Conditioning on $\mathcal{X}_{t^*}^{N_\kappa} = \mathcal{N}_\gamma$, by Lemma 3 ii) (with t^* replacing $t = 0$ as initial time),

$$\mathbb{E}_{\mathcal{X}_{t^*}^{N_\kappa}}\langle \mathcal{X}_t^{N_\kappa}, f\rangle \to \langle \mathcal{N}_\gamma, T_{t-t^*}^{\gamma,\kappa} f\rangle$$
$$+\int_0^1 (f(y) - T_{t-t^*}^{\gamma,\kappa} f(y))\,\omega_{\gamma,\kappa}\psi_{\gamma,\kappa}(y)\,dy + f(1)\,\omega_{\gamma,\kappa}\,(t-t^*).$$

By Lemma 3 iii),

$$\mathbb{E}\,\langle \mathcal{N}_\gamma, T_{t-t^*}^{\gamma,\kappa} f\rangle \to \int_0^1 T_{t-t^*}^{\gamma,\kappa} f(y)\,\omega_{\gamma,1}\psi_{\gamma,1}(y)\,dy + T_{t-t^*}^{\gamma,\kappa} f(1)\omega_{\gamma,1}\,t^*. \tag{IV}$$

Since $T_t^{\gamma,\kappa} f(1) = f(1)$ for each t, it follows that

$$\mathbb{E}\langle \mathcal{X}_t^{N_\kappa}, f\rangle \to \int_0^1 T_{t-t^*}^{\gamma,\kappa} f(y)\,\omega_{\gamma,1}\psi_{\gamma,1}(y)\,dy + f(1)\omega_{\gamma,1}\,t^*$$
$$+\int_0^1 (f(y) - T_{t-t^*}^{\gamma,\kappa} f(y))\,\omega_{\gamma,\kappa}\psi_{\gamma,\kappa}(y)\,dy + f(1)\,\omega_{\gamma,\kappa}\,(t-t^*),$$

which is the desired relation and hence completes the proof. □

We make two remarks: for suitable functions f with the additional property $f(1) = 0$, the nonstationary AFS interpolates between the two marginal limit spectra at time $t = t^*$ and as $t \to \infty$,

$$\mathbb{E}\,\langle \mathcal{X}_{t^*}^{N_\kappa}, f\rangle = \theta\int_0^1 f(y)\omega_{\gamma,1}\psi_{\gamma,1}(y)dy \quad \text{and} \quad \mathbb{E}\,\langle \mathcal{X}_\infty^{N_\kappa}, f\rangle \to \theta\int_0^1 f(y)\omega_{\gamma,\kappa}\psi_{\gamma,\kappa}(y)dy.$$

Both these spectra are stationary for population size equal to N and κN, respectively. For the case of neutral evolution, $\gamma = 0$, it holds $\omega_{0,1}\psi_{0,1}(y) - \omega_{0,\kappa}\psi_{0,\kappa}(y) = 2(1-\kappa)/y$ and hence, for suitable functions f with $f(1) = 0$, $t \geq t^*$, and $N \to \infty$,

$$\mathbb{E}\,\langle \mathcal{X}_t^{N_\kappa}, f\rangle \to 2\theta(1-\kappa)\int_0^1 y^{-1}\mathbb{E}_y^{0,\kappa}[f(\xi_t^{t^*})]\,dy + 2\theta\kappa\int_0^1 y^{-1}f(y)\,dy + \theta f(1)t.$$

1.3. Nucleotide diversity

For computing nucleotide diversity, we apply the AFS to the function $f_{\mathrm{pw}}(y) = 2y(1-y)$ and note that $f_{\mathrm{pw}}(1) = 0$. For the neutral case it holds in equilibrium according to Eq. (5)

$$\pi_S^\kappa = \theta\int_0^1 2y(1-y)\omega_{0,\kappa}\psi_{0,\kappa}(y)\,dy = 4\theta\kappa\int_0^1 (1-y)\,dy = 2\theta\kappa,$$

since $\omega_{0,\kappa} = 1$ and $\psi_{0,\kappa} = 2\kappa/y$. The synonymous diversity during nonequilibrium is obtained by applying the AFS in Eq. (6) to $f_{\mathrm{pw}}(y)$,

$$\begin{aligned}\pi_S^\kappa(t) &= \theta\int_0^1 \mathbb{E}_y^{0,\kappa}[2\xi_t^{t^*}(1-\xi_t^{t^*})]\Big(\frac{2}{y} - \frac{2\kappa}{y}\Big)\,dy + \theta\int_0^1 2y(1-y)\frac{2\kappa}{y}\,dy \\ &= 2\theta(1-\kappa)e^{-(t-t^*)/\kappa} + 2\theta\kappa,\end{aligned}$$

where we used Itô's formula to compute $\mathbb{E}_y^{0,\kappa}[\xi_t^{t^*}(1-\xi_t^{t^*})] = e^{-(t-t^*)/\kappa}y(1-y)$. It holds $\pi_S^\kappa(t^*) = 2\theta$ and $\pi_S^\kappa(t) = \pi_S^\kappa = 2\theta\kappa$ in the limit $t \to \infty$.

For nonsynonymous nucleotide differences we proceed similarly but use the DFE in Eq. (12) to allow for variation in selection. The stationary case follows by integration of Eq. (11) over the DFE,

$$\mathbb{E}\pi_N^{\mathcal{V},\kappa} = 4\theta\kappa\int_{-\infty}^0 \Big(\frac{1}{1-e^{-2v\kappa}} - \frac{1}{2v\kappa}\Big)h_{\mathcal{V}}(v)\,dv.$$

This integral is well-defined, which can be seen by looking at the Taylor expansion of the integrand around zero and noting that the moments of the gamma distribution are finite.

For the behavior of π_N in nonequilibrium we apply the nonstationary AFS stated in Eq. (6) to $f_{\mathrm{pw}}(y)$, i.e.

$$\begin{aligned}\pi_N^\kappa(t) &= \theta \int_{-\infty}^0 \int_0^1 \mathbb{E}_y^{v,\kappa}[2\xi_t^{t^*}(1-\xi_t^{t^*})](\omega_{v,1}\psi_{v,1}(y) - \omega_{v,\kappa}\psi_{v,\kappa}(y))h_{\mathcal{V}}(v)\,dy\,dv \\ &\quad + \theta \int_{-\infty}^0 \int_0^1 2y(1-y)\omega_{v,\kappa}\psi_{v,\kappa}(y)h_{\mathcal{V}}(v)\,dy\,dv \\ &= 2\theta\mathbb{E}\int_0^1 \mathbb{E}_y^{\mathcal{V},\kappa}[\xi_t^{t^*}(1-\xi_t^{t^*})](\omega_{\mathcal{V},1}\psi_{\mathcal{V},1}(y) - \omega_{\mathcal{V},\kappa}\psi_{\mathcal{V},\kappa}(y))\,dy + \mathbb{E}\pi_N^{\mathcal{V},\kappa}.\end{aligned}$$

Since $\xi_t^{t^*} \in \{0,1\}$ for $t \to \infty$, it holds $\mathbb{E}_y^{\mathcal{V},\kappa}[\xi_t^{t^*}(1-\xi_t^{t^*})] = 0$ in the limit $t \to \infty$, implying $\pi_N^\kappa(t) \to \mathbb{E}\pi_N^{\mathcal{V},\kappa}$. We can further explicitly write

$$\begin{aligned}&\omega_{\gamma,1}\psi_{\gamma,1}(y) - \omega_{\gamma,\kappa}\psi_{\gamma,\kappa}(y) \\ &\quad = \frac{2}{y(1-y)}\Big(\frac{1-e^{-2\gamma(1-y)}}{1-e^{-2\gamma}} - \frac{\kappa(1-e^{-2\gamma\kappa(1-y)})}{1-e^{-2\gamma\kappa}}\Big)\end{aligned} \tag{V}$$

for $\gamma \neq 0$, and $\psi_{0,1}(y) - \psi_{0,\kappa}(y) = 2/y - 2\kappa/y = 2(1-\kappa)/y$.

1.4. Number of fixations

The number of fixations before the change in population size, i.e. $Z^{\gamma,\kappa}(t)$ for $t \leq t^*$, is obtained by applying the equilibrium AFS in Eq. (5) to $f_{\mathrm{fix}}(y)$,

$$Z^{\gamma,\kappa}(t) = \theta\,\omega_{\gamma,1}\,f_{\mathrm{fix}}(1)\,t + \theta\int_0^1 f_{\mathrm{fix}}(y)\omega_{\gamma,1}\psi_{\gamma,1}(y)\,dy = \theta\omega_{\gamma,1}t.$$

The integral term vanishes and $\kappa = 1$, since for $t \leq t^*$ the population size is $N_\kappa = N$.

For $t > t^*$, we apply the nonequilibrium AFS in Eq. (6),

$$\begin{aligned}Z^{\gamma,\kappa}(t) &= \theta\{\omega_{\gamma,1}\,t^* + \omega_{\gamma,\kappa}\,(t-t^*)\} \\ &\quad + \theta\int_0^1 \mathbb{E}_y^{\gamma,\kappa} f_{\mathrm{fix}}(\xi_t^{t^*})\,(\omega_{\gamma,1}\psi_{\gamma,1}(y) - \omega_{\gamma,\kappa}\psi_{\gamma,\kappa}(y))\,dy \\ &\quad + \theta\int_0^1 f_{\mathrm{fix}}(y)\,\omega_{\gamma,\kappa}\psi_{\gamma,\kappa}(y)\,dy.\end{aligned}$$

Since the last term vanishes and $\mathbb{E}_y^{\gamma,\kappa} f_{\text{fix}}(\xi_t^{t^*}) = \mathbb{P}_y^{\gamma,\kappa}(\tau_1 \leq t - t^*)$, as we have shown in Eq. (15), it follows

$$Z^{\gamma,\kappa}(t) = \theta\{\omega_{\gamma,1}\, t^* + \omega_{\gamma,\kappa}\,(t - t^*)\} \\ + \theta \int_0^1 \mathbb{P}_y^{\gamma,\kappa}(\tau_1 \leq t - t^*)\,(\omega_{\gamma,1}\psi_{\gamma,1}(y) - \omega_{\gamma,\kappa}\psi_{\gamma,\kappa}(y))\,dy.$$

The explicit representation for $\omega_{\gamma,1}\psi_{\gamma,1}(y) - \omega_{\gamma,\kappa}\psi_{\gamma,\kappa}(y)$ is given in Eq. (V).

References

Kaj, I. and Mugal, C. F. 2016. The non-equilibrium allele frequency spectrum in a Poisson random field framework. *Theor. Popul. Biol.*, 111: 51–64.

2. Supplemental Figures

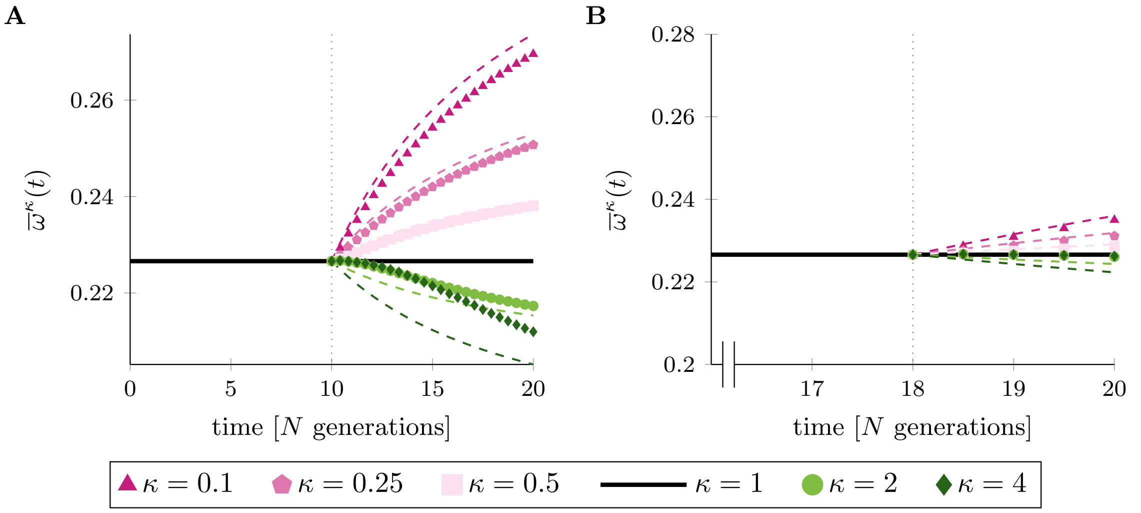

Fig. S1: The fixation rate ratio $\overline{\omega}^{\kappa}(t)$ for different values of κ as functions of time. Panel A: change in population size at time $t^* = 10$. Panel B: change in population size at time $t^* = 18$. For comparison, colored, dashed curves represent the weighted fixation rate ratio $\omega_{\mathrm{w}}^{\kappa}(t)$. Vertical, dotted lines indicate time t^*. Parameters: $\theta = 1$, and $a = 0.15$ and $ab = 2500$ for the DFE.
