# Supplementary figures and images for: A Nearly Neutral Model of Molecular Signatures of Natural Selection after Change in Population Size
Source: Genome Biol Evol. 2022 Apr 27;14(5):evac058. doi: 10.1093/gbe/evac058 (PMC9127441; doi:10.1093/gbe/evac058)

**A**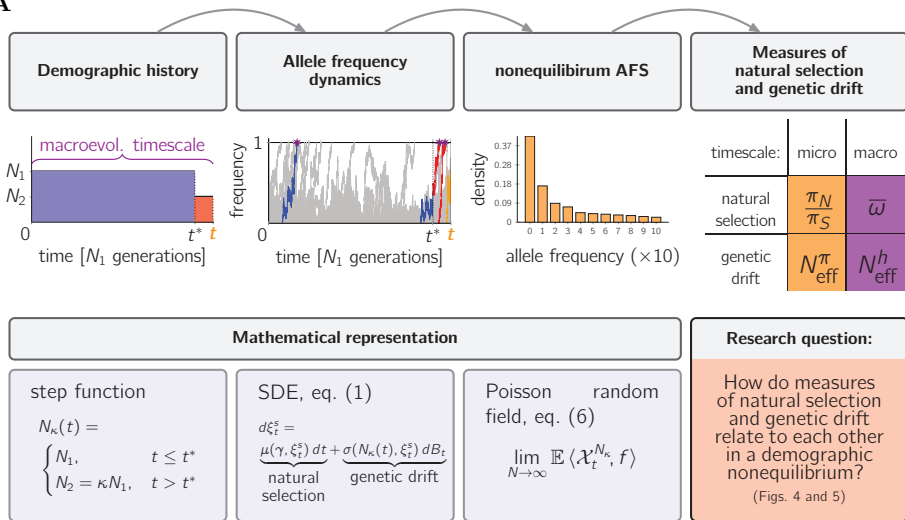**B**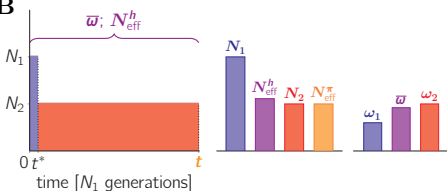**C**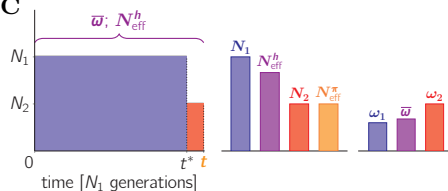

Supplement: evac058_Supplementary_Data [file evac058_supplementary_data.zip › fig1.pdf]

**A**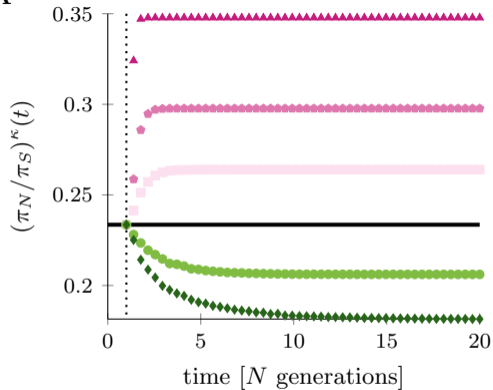**B**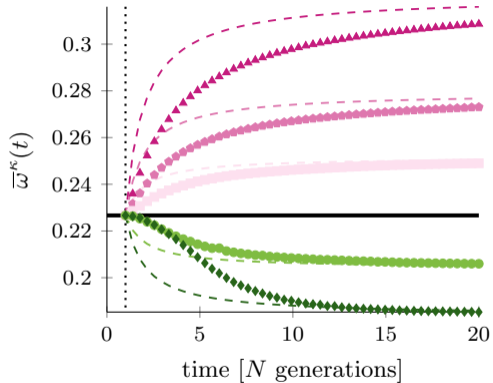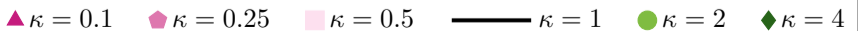

Supplement: evac058_Supplementary_Data [file evac058_supplementary_data.zip › fig2.pdf]

**A**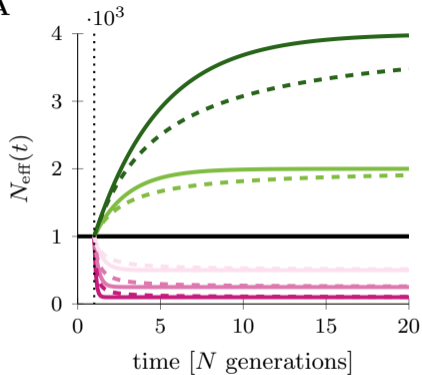**B**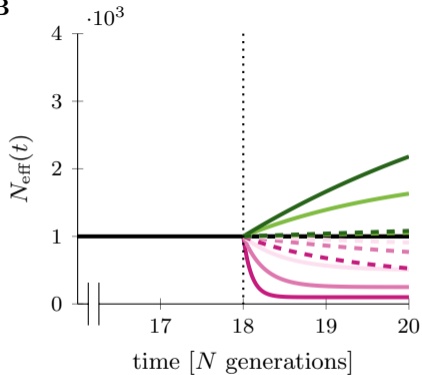

$\kappa = 0.1$     $\kappa = 0.25$     $\kappa = 0.5$     $\kappa = 1$     $\kappa = 2$     $\kappa = 4$

Supplement: evac058_Supplementary_Data [file evac058_supplementary_data.zip › fig3.pdf]

A

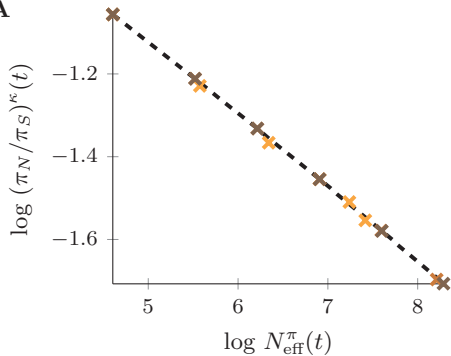

B

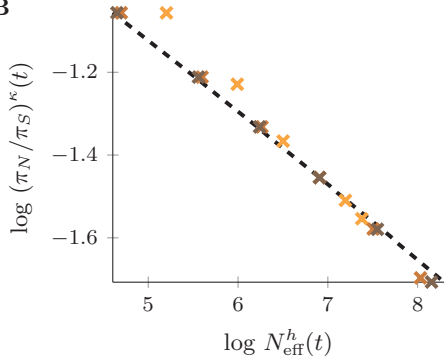

C

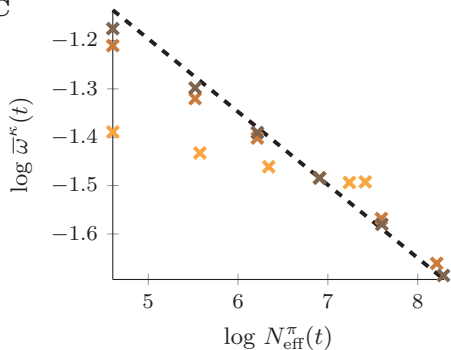

D

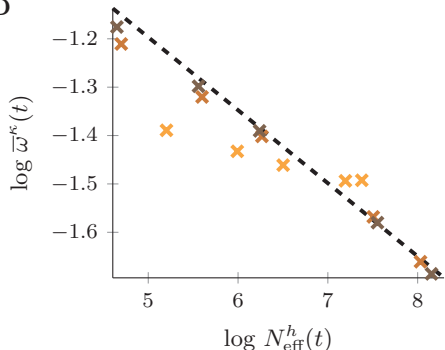

----- equilibrium    ×  $t=2$     ×  $t=10$     ×  $t=20$

Supplement: evac058_Supplementary_Data [file evac058_supplementary_data.zip › fig4.pdf]

A

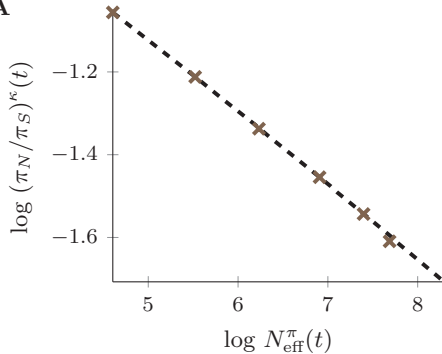

B

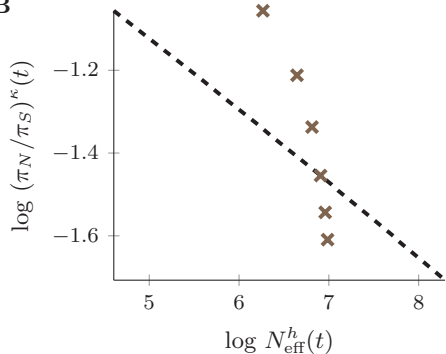

C

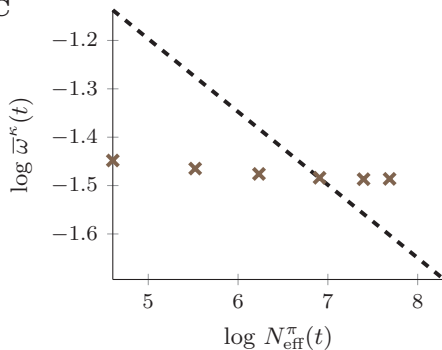

D

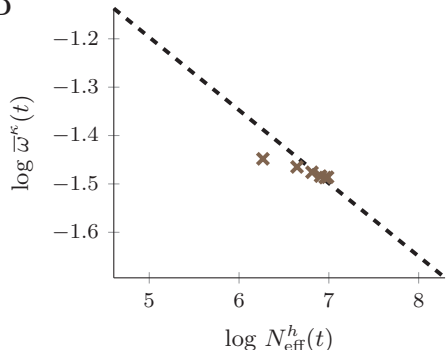

----- equilibrium    x  $t = 20$

Supplement: evac058_Supplementary_Data [file evac058_supplementary_data.zip › fig5.pdf]
